# Structural and functional probing of PorZ, an essential bacterial surface component of the type-IX secretion system of human oral-microbiomic *Porphyromonas gingivalis*.

**DOI:** 10.1038/srep37708

**Published:** 2016-11-24

**Authors:** Anna M. Lasica, Theodoros Goulas, Danuta Mizgalska, Xiaoyan Zhou, Iñaki de Diego, Mirosław Ksiazek, Mariusz Madej, Yonghua Guo, Tibisay Guevara, Magdalena Nowak, Barbara Potempa, Apoorv Goel, Maryta Sztukowska, Apurva T. Prabhakar, Monika Bzowska, Magdalena Widziolek, Ida B. Thøgersen, Jan J. Enghild, Mary Simonian, Arkadiusz W. Kulczyk, Ky-Anh Nguyen, Jan Potempa, F. Xavier Gomis-Rüth

**Affiliations:** 1 Department of Oral Immunology and Infectious Diseases, University of Louisville School of Dentistry, Louisville, KY, USA; 2 Department of Bacterial Genetics, Institute of Microbiology, Faculty of Biology, University of Warsaw, Warsaw, Poland; 3 Proteolysis Lab, Structural Biology Unit (“María-de-Maeztu¨ Unit of Excellence), Molecular Biology Institute of Barcelona (CSIC), Barcelona Science Park, Barcelona, Catalonia, Spain; 4 Department of Microbiology, Faculty of Biochemistry, Biophysics, and Biotechnology, Jagiellonian University, Krakow, Poland; 5 Department of Oral Biology, Faculty of Dentistry, University of Sydney, Sydney, NSW 2006, Australia; 6 Department of Cell Biochemistry, Faculty of Biochemistry, Biophysics, and Biotechnology, Jagiellonian University, Krakow, Poland; 7 Interdisciplinary Nanoscience Center (iNANO), and the Department of Molecular Biology, Aarhus University, Århus, DK-8000, Denmark; 8 Institute of Dental Research, Westmead Centre for Oral Health and Westmead Institute for Medical Research, Sydney, NSW 2145, Australia; 9 Department of Biological Chemistry and Molecular Pharmacology, Harvard University Medical School, Boston, MA, USA; 10 Małopolska Center of Biotechnology, Jagiellonian University, Krakow, Poland

## Abstract

*Porphyromonas gingivalis* is a member of the human oral microbiome abundant in dysbiosis and implicated in the pathogenesis of periodontal (gum) disease. It employs a newly described type-IX secretion system (T9SS) for secretion of virulence factors. Cargo proteins destined for secretion through T9SS carry a recognition signal in the conserved C-terminal domain (CTD), which is removed by sortase PorU during translocation. Here, we identified a novel component of T9SS, PorZ, which is essential for surface exposure of PorU and posttranslational modification of T9SS cargo proteins. These include maturation of enzyme precursors, CTD removal and attachment of anionic lipopolysaccharide for anchorage in the outer membrane. The crystal structure of PorZ revealed two β-propeller domains and a C-terminal β-sandwich domain, which conforms to the canonical CTD architecture. We further documented that PorZ is itself transported to the cell surface *via* T9SS as a full-length protein with its CTD intact, independently of the presence or activity of PorU. Taken together, our results shed light on the architecture and possible function of a novel component of the T9SS. Knowledge of how T9SS operates will contribute to our understanding of protein secretion as part of host-microbiome interactions by dysbiotic members of the human oral cavity.

As part of host-microbiome interactions, resident bacteria secrete proteins, lipopolysaccharides, and peptidoglycan into the extracellular environment to facilitate antibiotic resistance, deterrence of host immune defenses, attachment, detoxification and nutrient acquisition. This helps them to flourish in a densely populated, highly competitive environment^1^,^2^. In diderm prokaryotes, represented mostly by Gram-negative bacteria, secreted proteins are synthesized in the cytoplasm and then translocated across two lipid bilayers: the inner (IM) and outer (OM) membranes, which have a periplasmic space between them. To achieve this, diderm bacteria have so far evolved nine known types (I to IX) of secretion systems (TxSS)^1^,^2^,^3^. Using such systems, they can assemble cell-surface appendages such as pili (mediated by T2SS, T4SS and T7SS), curli (T8SS), and flagella (T3SS); secrete proteins to the extracellular space (T1SS to T6SS); and inject proteins into eukaryotic host cells (T3SS and T4SS) or the periplasm of other bacteria (T6SS). Proteins to be translocated are either directly secreted from the cytoplasm *via* T1SS, T3SS, T4SS and T6SS or are first exported to the periplasm across the IM using conserved *Sec* or *Tat* pathways and then translocated through the OM using T2SS, T5SS, T7SS or T8SS^3^. The most recently discovered system of protein secretion is T9SS, also called *Por secretion system*, which operates exclusively in selected species within the Bacteroidetes phylum^4^,^5^,^6^,^7^,^8^,^9^. The Bacteroidetes and Firmicutes phyla accounts for +99% of species and phylotypes of the human gut microbiome^10^. In the oral microbiome, members that colonize dental plaque in periodontal disease such as *Porphyromonas gingivalis* and *Tannerella forsythia* also possess T9SS. These bacteria dominate the dysbiotic biofilm when the commensal microbiome is disrupted. Together with *Treponema denticola*, they give rise to the “red complex” microbial consortium commonly found in severe periodontal disease^11^. T9SS is essential for the secretion of many proteinaceous virulence factors by *P. gingivalis*, and has, thus, attracted considerable attention as a possible target for pharmaceutical intervention to treat severe periodontal disease and restore homeostasis of the oral microbiome^4^,^12^.

All cargo proteins of *P. gingivalis* T9SS contain a conserved C-terminal domain (CTD) of ~70 residues required for export and attachment to the cell surface^13^,^14^,^15^, which was recently shown to adopt an antiparallel seven-stranded immunoglobulin-like architecture^12^. T9SS cargo proteins carry a typical cleavable N-terminal signal peptide for export across the IM to the periplasm using the *Sec* system. Proteins fold in the periplasm, and are then directed to the T9SS translocon by a targeting signal located in the last two β-strands of CTD^12^,^15^. During this translocation, CTD is cleaved off^16^ and the protein is released extracellularly^17^. At least in selected proteins of *P. gingivalis*, CTD removal occurs concurrently with covalent attachment of anionic lipopolysaccharide (A-LPS) to the newly released C-terminal carboxylate of the processed protein^16^. A-LPS attachment serves to anchor secreted proteins to the OM where they form an electron-dense surface layer, which is characteristic of *P. gingivalis*^18^.

To date, 12 indispensable T9SS genes have been identified in *P. gingivalis*^8^. These are conserved across species that have T9SS, which suggests that they are generally required for assembly or functioning of the secretory apparatus^5^. Among the translated products of these genes, five are predicted to be integral-membrane β-barrels located in the OM (PorP, PorQ, PorT, PorV and Sov); two are type-I inner-membrane proteins (PorM and PorL); and three are putative lipoproteins (PorK, PorW and the unnamed *PG1058* gene product). Recently, lipoproteins PorN and the PorK were shown to interact and form a ring-shaped structure 50-nm in diameter, which is anchored on the periplasmic side of the OM as an integral component of the T9SS machinery^19^. Finally, PorU is found on the bacterial surface with an intact CTD^16^,^17^. In *P. gingivalis*, inactivation of any of these components leads to the arrest of T9SS cargo proteins in the periplasm, with an intact CTD^4^. Apart from PorU, which is a surface-located cysteine peptidase that functions as a sortase to cleave off the CTD from secreted proteins^18^, little is known about the functional or structural roles of the other T9SS components.

*P. gingivalis* encodes 32 putative CTD-containing proteins, which include PorU and important virulence factors such as the gingipain cysteine peptidases RgpA, RgpB, and Kgp^20^,^21^; carboxypeptidase D (*alias* Cpg70^22^); 35-kDa heme-binding protein (HBP35^23^,^24^) and peptidylarginine deiminase (PPAD^25^,^26^). All of these proteins (apart from PorU which uses LptO/PorV as an anchor) have been shown to be A-LPS-modified after secretion: when they were recovered from the cell envelope, OM or OM vesicles, they migrated in SDS-PAGE with a higher molecular mass than predicted, and reacted to A-LPS-specific antibodies. This indicates that they are secreted and glycosylated *via* T9SS^27^. In contrast, the protein product of gene *PG1604* (also known as *PG_RS07070*), tentatively annotated as immunoreactive OM-associated 84-kDa antigen PG93 (see UniProt [UP] database entry Q9S3Q8), was shown in proteomic studies to retain an intact CTD upon secretion^27^,^28^. Moreover, putative homologues of this protein were found in other Bacteroidetes with T9SS (*Prevotella intermedia*, *Parabacteroides distasonis* and *Cytophaga hutchinsonii*^27^). An intact CTD is inconsistent with T9SS cargos, but rather reminiscent of intrinsic T9SS component PorU. Thus, the *PG1604* gene product was hypothesized to be a new, conserved component of the T9SS machinery. To verify this hypothesis, we probed the function of the protein by targeted mutagenesis directly in *P. gingivalis*, and analysed the effect of the isogenic *PG1604* gene deletion on T9SS cargo transcription and secretion. In addition, we determined the X-ray crystal structure of the protein to assess the molecular determinants of its function. Cumulatively, the results indicated unambiguously that the *PG1604* product is an essential component of T9SS. To be consistent with the nomenclature of T9SS components, we suggest to call it PorZ.

## Results and Discussion

### PorZ is an essential component of T9SS

**Figure 1:**
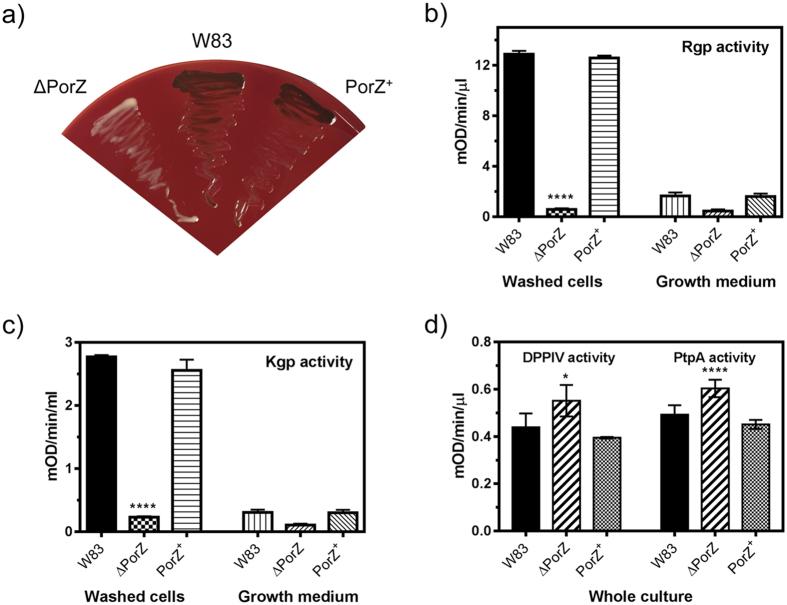
Characterization of the ΔPorZ secretory phenotype. (**a**) Pigmentation on blood agar of *P. gingivalis* wild type (W83), ΔPorZ, and *in trans porZ-*complemented ΔPorZ (PorZ^+^) strains. Enzymatic activity of (**b**) Rgps, (**c**) Kgp, and (**d**) dipeptidyl peptidase IV (DPPIV) and prolyl tripeptidyl peptidase (PtpA) in whole cultures, fractionated washed cells or growth medium as determined with specific synthetic substrates. Cultures were adjusted to OD_600_ = 1.0 prior to testing and processing, and results shown correspond to triplicate experiments. Significant differences between the wild type and mutants are indicated by *P < 0.05 and ****P < 0.0001.

An isogenic deletion mutant of the *porZ* gene, ΔPorZ, was created by homologous recombination to assess its effect on T9SS cargo secretion and posttranslational processing. Deletion had a negligible effect on the *P. gingivalis* growth rate in complex media (Supplementary Fig. S1). However, on blood agar, the mutant could not accumulate heme on the cell surface and therefore yielded non-pigmented colonies (Fig. 1a). This is attributable to gingipains, which are secreted T9SS cargos that are essential for hemoglobin degradation and heme recruitment^29^,^30^,^31^. Therefore, lack of pigmentation in ΔPorZ suggested failure of functional gingipain secretion and activation. Indeed, we found that ΔPorZ was deficient in extracellular Kgp and Rgp gingipain activities when compared to the wild-type *P. gingivalis* strain W83 (hereafter, “wild type”; Fig. 1b,c). In contrast, dipeptidyl peptidase IV and prolyl tripeptidyl peptidase A, which are surface enzymes but not secreted by T9SS, were produced and transported to the bacterial surface in significantly higher amounts than in the wild type (Fig. 1d). A similar response had been previously observed in an inactivation mutant of an essential T9SS component, PorT^32^. This presumably reflects general upregulation of peptidolytic enzymes as a response to the absence of functional gingipains, which account for 85% of the extracellular proteolytic activity of *P. gingivalis*^33^. Reconstitution *in trans* of the *porZ* gene in the ΔPorZ mutant—yielding PorZ^+^—restored both pigmentation and proteolytic activity to wild-type levels (Fig. 1a–d).

**Figure 2:**
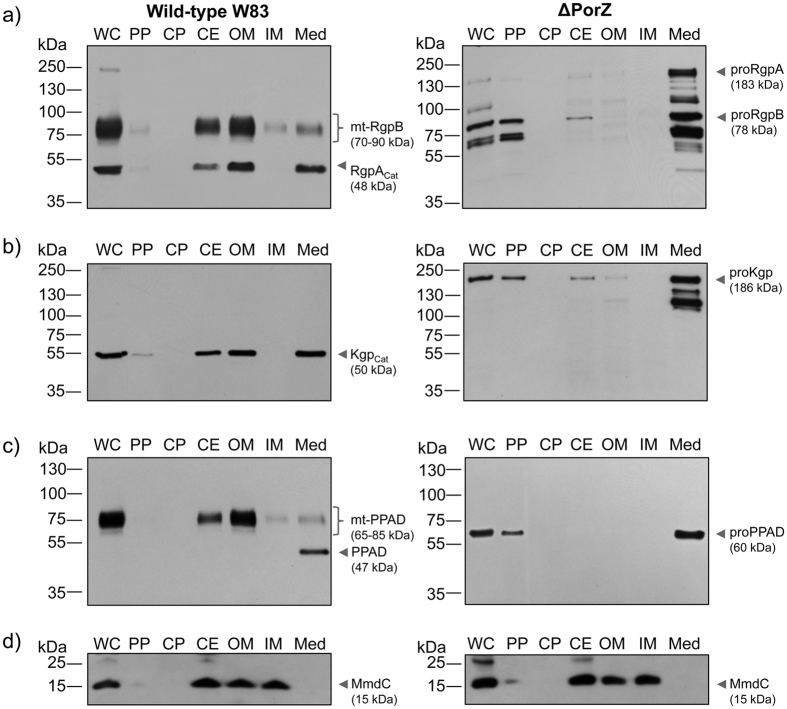
Subcellular location of gingipains and PPAD. Whole cells (WC) of wild-type (W83; *left panel*) and ΔPorZ (*right panel*) *P. gingivalis* strains were proportionately fractionated into periplasm (PP), cytoplasm (CP), cell envelope (CE), outer membrane (OM), inner membrane (IM) and culture medium fractions (Med; 10-fold concentrated); and probed for (**a**) Rgps, (**b**) Kgp, (**c**) PPAD by Western blotting with specific monoclonal antibodies and (**d**) biotinylated IM protein (MmdC) through reaction with streptavidin conjugated to horseradish peroxidase. The pinpointed and labeled bands correspond to: (**a**) catalytic domain of RgpA (RgpA_cat_) and membrane-type RgpB (mt-RgpB) in the wild type (*left panel*) and unprocessed pro-RgpA and pro-RgpB in ΔPorZ (*right panel*); (**b**) catalytic domain of Kgp (Kgp_cat_) in the wild type (*left panel*) and unprocessed pro-Kgp in ΔPorZ (*right panel*); (**c**) mature PPAD and membrane-type PPAD (mt-PPAD) in the wild type (*left panel*) and unprocessed pro-PPAD in ΔPorZ (*right panel*); and (**d**) MmdC in the wild-type (*left panel*) and ΔPorZ (*right panel*) strains.

To further investigate the fate of non-secreted T9SS cargos in the absence of PorZ, we performed Western blot analysis of distinct subcellular fractions to detect gingipains (Fig. 2a,b), PPAD (Fig. 2c), and the biotin-containing 15-kDa biotin carboxyl carrier protein (AccB *alias* MmdC or PG1609) as an IM marker (Fig. 2d; see also ref. 32). The latter analysis revealed that the OM fractions obtained from the wild type and the mutant were contaminated with the IM. This is in contrast to undetectable contamination of the IM fraction with OM components, as indicated by the absence of gingipains and PPAD in the IM fraction. In the wild type, gingipains and PPAD were secreted onto the cell surface with CTD removal and proteolytic maturation of their precursors, which led to detectable activity in intact cells^34^. In ΔPorZ, they were not processed to the mature forms but rather accumulated as precursors in the periplasmic fraction and in clarified culture media (Fig. 2a–c). Additionally, partially processed gingipain precursors were found with anti-gingipain antibodies. Auto-processing of gingipains has been described in heterologously-overexpressed recombinant proteinases^35^. Moreover, the presence of considerable amounts of PPAD and gingipain precursors in the concentrated growth medium suggested that ΔPorZ had a “leaky” OM architecture. This contention was supported by peptide mass fingerprinting of proteins from the growth medium resolved on SDS-PAGE (Supplementary Fig. S2). Although we found in the medium several proteins normally located in the periplasm, including prolyl oligopeptidase family proteins (PG0727 and PG1004), a MEROPS-M16-family peptidase (PG0196), thioredoxin (PG0275), HtrA protease/chaperone (PG0449) and TPR-domain protein (PG0449), CTD-bearing proteins were predominant. Indeed, of the 32 known T9SS cargos of *P. gingivalis*^28^, 12 were found in high abundance and apparently with intact CTDs in the growth medium of ΔPorZ, as indicated by high Mascot scores (Supplementary Table S1). These proteins included PorU (*alias* PG0026), carboxypeptidase D (*alias* Cpg70 or PG0232), PPAD (PG1424), internalin-like protein PG0350, putative hemagglutinin PG0411, immunoreactive 47-kDa antigen PG97 (PG1374), immunoreactive 46-kDa antigen PG99 (PG1798), heme-binding protein 30 (PG0616), and proteins PG0495, PG0654, PG1030, and PG2216. In addition, five other potential T9SS cargos were found in the medium, but none of the detected peptides corresponded to their CTDs. When the same proteins were detectable in the growth medium of the wild-type strain, they had much lower Mascot scores and no peptides corresponding to their respective CTDs. This “leaky” OM phenotype, which leads to release of non-cleaved CTDs from CTD-cargo proteins, is similar to that reported by Taguchi *et al.*^36^. These authors reported that the chaperone Skp-like protein (PGN_0300) is required for OM insertion of PorU sortase, which in turn is necessary for CTD cleavage from CTD-cargo proteins. Consistently, deletion of PGN_0300 resulted in failure of PorU insertion and, thus, T9SS function^36^.

### PorZ is located on the cell surface of *P. gingivalis*

**Figure 3:**
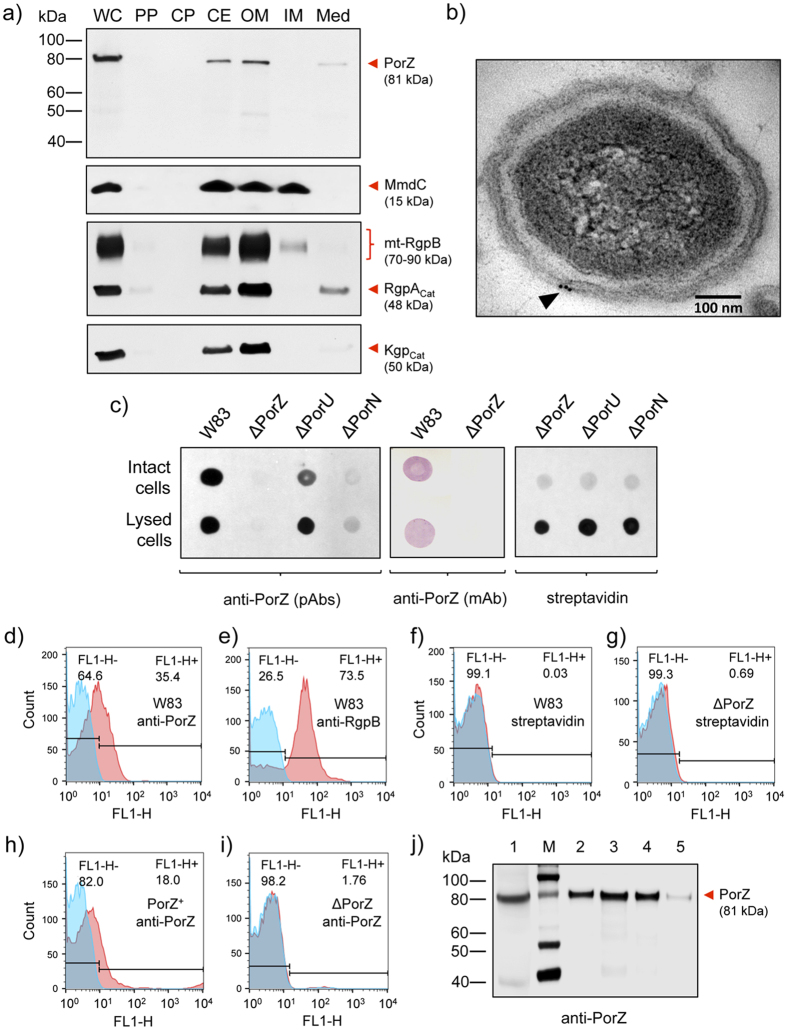
PorZ is located on the cell surface of *P. gingivalis*. (**a**) Wild-type *P. gingivalis* cells (W83) were proportionately fractionated into whole cell extract (WC), periplasm (PP), cytoplasm (CP), cell envelope (CE), outer membrane (OM), inner membrane (IM) and growth medium (Med), and subsequently analyzed by Western blotting using mouse polyclonal anti-PorZ and anti-Rgp antibodies and mouse monoclonal anti-Kgp antibodies. Streptavidin conjugated to horseradish peroxidase was used to detect MmdC, a biotinylated IM-associated protein. Presence or absence of full-length PorZ (81-kDa band) and other proteins is indicated. (**b**) Wild-type cells were probed with monoclonal anti-PorZ antibodies and labeled with immunogold to visualize the cellular location of PorZ (*black arrowhead*) in electron microscopy (bar = 100 nm). (**c**) Dot blot analysis of intact and lyzed wild-type (W83), ΔPorZ, ΔPorU and ΔPorN cells using mouse monoclonal anti-PorZ antibodies (mAb), mouse polyclonal anti-PorZ antibodies (pAb) or streptavidin conjugated to horseradish peroxidase. Flow cytometry analysis showing the surface exposure of **(d)** PorZ in wild-type cells (W83) with anti-PorZ pAb; **(e)** RgpB in wild-type cells (W83) with monoclonal anti-RgpB antibodies (positive control); **(f)** MmdC in wild-type cells (W83) and (**g**) in ΔPorZ cells with streptavidin-Alexa Fluor 488 conjugate**; (h)** PorZ in *in trans porZ*-complemented ΔPorZ (PorZ^+^) cells with pAb and **(i)** PorZ in ΔPorZ cells with anti-PorZ pAb. Isotype negative controls are in blue and immunoprobed cells in red; the histograms shown are representative of three independent experiments. (**j**) Presence of full-length PorZ (*lane 1*) detected by Western blot using anti-PorZ pAb on wild-type cells washed with PBS and suspended, respectively, in distilled water (*lane2*); in 0.0007% Tween-20 (*lane 3*); in 0.04% sarcosyl (*lane 4*); and in 0.02% SDS (*lane 5*). After 10 min of gentle stirring, cells were removed by centrifugation and the presence of PorZ in the cell pellet (*lane 1*) and supernatants (*lanes 2–5* was checked). The detergent concentrations correspond to one-tenth of the critical micelle concentration (CMC). *Lane M*, MagicMark™ XP Western Protein Standard.

Previous proteomics studies identified PorZ in the OM and OM vesicles (OMV) of *P. gingivalis* strain W50^27^,^28^. To determine the location of the protein more precisely, we performed Western blot analysis on wild-type cultures, quantitatively separated into whole cells and sub-cellular fractions, which included growth medium, periplasm, cytoplasm, and the cell envelope (IM+OM). The latter was additionally fractionated with detergent into OM and IM fractions. Fraction purity was verified by Western blot using Rgp/Kgp and MmdC as markers for the OM and IM, respectively. Again, while the IM fraction was only very slightly contaminated with the OM, the latter fractions contained a notable amount of the IM, as indicated by the presence of MmdC in these fractions (Fig. 3a). In agreement with the predicted localization, PorZ was mostly found associated with the cell envelope and OM fractions (Fig. 3a), as confirmed by immunogold-staining electron microscopy (Fig. 3b). In addition, trace amounts of the PorZ protein were detected in concentrated growth medium together with RgpA_cat_ and Kgp_cat_, suggesting that, similarly to gingipain catalytic domains, also PorZ can be shed in low amounts from the bacterial surface into the medium.

To determine whether PorZ is located on the periplasmic or the extracellular side of the OM, we performed dot-blot analysis of intact and sonicated wild-type cells *plus* ΔPorZ as a negative control. While no signal was detected for the latter, equivalent staining was observed for both intact and sonicated cells employing two different anti-PorZ antibodies (Fig. 3c). Of note, the integrity of the *P. gingivalis* cell envelope was confirmed by dot-blot analysis with streptavidin-HRP showing the reaction only after cell disruption by sonication. These findings support cell-surface localization of PorZ (Fig. 3c). This location was further assessed by flow cytometry, with surface-exposed gingipain RgpB and intracellular MmdC as positive and negative controls, respectively. Indeed, when anti-PorZ and anti-RgpB antibodies were used, both proteins were identified on the cell surface (Fig. 3d,e), while no staining was detected with streptavidin-Alexa Fluor 488 conjugate (Fig. 3f,g). Specificity of the flow cytometry analysis was checked with ΔPorZ, which showed negligible staining, while a strong signal was found in the *in trans porZ*-complemented strain, PorZ^+^ (Fig. 3h,i). Interestingly, FACS assays with *P. gingivalis* strain HG66, which cannot attach A-LPS to T9SS cargos so that these are subsequently released into the extracellular milieu, revealed that PorZ was still on the cell surface (Supplementary Fig. S3). Finally, when intact *P. gingivalis* cells were gently mixed with pure distilled water or subcritical micellar concentrations of detergents, which are unable to disrupt OM integrity, a sizable fraction of PorZ was released into the liquid phase (Fig. 3j). Significantly, only trace amounts of gingipains were washed out of the bacterial surface under the conditions tested (Supplementary Fig. S3).

### PorZ affects expression of T9SS components and cargos

**Figure 4:**
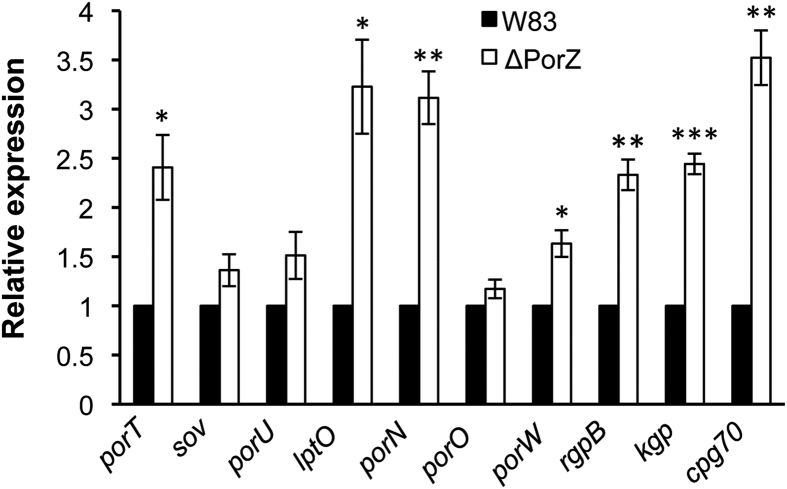
Effect of PorZ on the expression of T9SS components and cargos. The respective mRNAs of the genes of T9SS components (*porT*, *sov*, *porU*, *lptO*, *porN*, *porO* and *porW*) and cargos (*rgpB*, *kgp* and *cpg70*) were quantified in mid-logarithmic cultures of wild type (W83; full bars) and ΔPorZ (open bars) cells by quantitative RT-PCR. Expression levels were normalized against ribosomal *r16s* expression, and the respective expression levels of the wild type were arbitrarily set to 1.0. The results correspond to four independent experiments. Error bars represent standard deviation as analyzed by Student’s t-test. *P < 0.05; **P < 0.01 and ***P < 0.001.

To determine whether the absence of gingipain activity in the ΔPorZ mutant was also due to their reduced expression, quantitative RT-PCR was used to investigate expression of CTD-cargo proteins and other T9SS components in the ΔPorZ mutant. Interestingly, rather than suppression of CTD-cargo protein expression, the mRNA levels of CTD-cargo peptidases RgpB, Kgp and CPG70 in ΔPorZ were found to double or triple those of the wild type (Fig. 4). Thus, lack of activity was due to their failure to be secreted and maturated in ΔPorZ rather than to reduced expression. Further, deletion of PorZ induced significant upregulation of T9SS-components *porT*, *lptO*, and *porN* when compared to the wild type. In particular, the latter two triplicated the expression levels of the wild type (Fig. 4). Other components such as *porO*, *porW*, *sov* and *porU* did not show significant change. These trends were consistent regardless of whether *r16s* (Fig. 4) or *gyrA* (data not shown) was used as a reference gene. Overall, the significance of this variable effect on the expression of T9SS components in ΔPorZ is currently unknown.

### Structural analysis of PorZ

**Figure 5:**
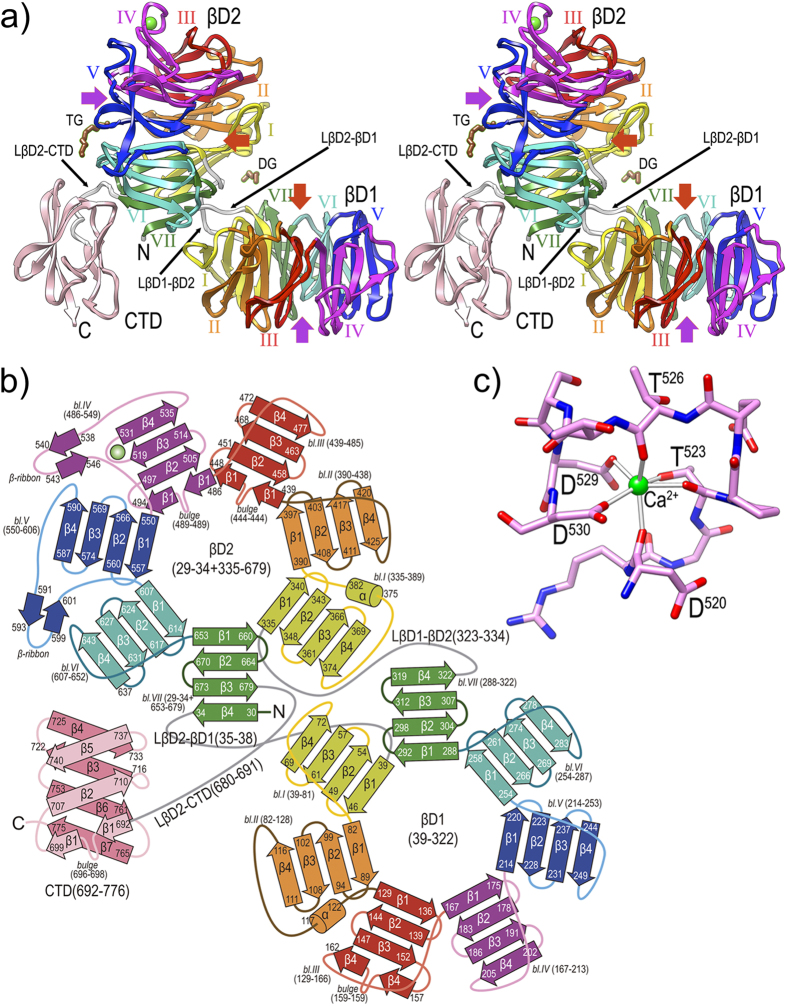
Overall crystal structure of PorZ. (**a**) Ribbon-type plot in cross-eye stereo of the crystal structure to 2.9 Å resolution of PorZ depicting domains βD1, βD2 and CTD, and the three domain-connecting linkers (white ribbons; labeled LβD2-βD1, LβD1-βD2, and LβD2-CTD). Each of the seven blades of propellers βD1 and βD2 (labeled counter-clockwise I to VII) is colored in yellow, orange, red, magenta, blue, turquoise and green, respectively; the CTD is in pink. A structural calcium-binding site (green sphere) is found within βD1-blade IV, and a tetraethylene glycol (TG) and a diethylene glycol (DG) were tentaively assigned on the protein surface (brown stick-models). Other (functionally probably irrelevant) ions and ligands were omitted for clarity. The central shafts of βD1 and βD2 are pinpointed on the entry and exit sides of the propellers by red and purple arrows, respectively. For labels and extension of regular secondary structure elements, see (b). (**b**) Topology scheme of PorZ, with β-strands as arrows and helices as cylinders, colored as in (a). The polypeptide chain spans residues G^29^—R^776^ and the three constituting domains *plus* the linkers (in grey) are indicated with the residues delimiting each structural element (strands, bulges, helices, β-ribbons, blades and domains). The nomenclature adopted in the text for structure elements is “domain-blade-structural element”, e.g. βD1-VI-β3 or βD2-IV-β-ribbon. **(c)** Structural calcium-binding site framed by segment D^520^—D^530^ within loop Lβ3β4 of βD1-blade IV. The ion is octahedrally coordinated by D^520^O, T^523^O, T^523^Oγ, T^526^O, D^529^Oδ1 and D^530^Oδ1, which are at binding distances of ~2.4 Å.

We produced PorZ without its predicted signal peptide (residues Q^26^-R^776^) by recombinant overexpression in *Escherichia coli*, and succeeded in crystallizing and solving its structure by single-wavelength anomalous diffraction with a selenomethionine derivative. The structure was refined with data to 2.9 Å resolution and consists of three domains. The first two are consecutive N-terminal seven-stranded β-propeller or circular-leaflet moieties (βD1: residues K^39^—M^322^; and βD2: G^29^—L^34^+Y^335^—T^679 ^37,38,39; PorZ residue numbering in superscript notation according to UP Q9S3Q8), each featuring a shallow cylinder or thick disk with an “entry side” and an “exit side”^38^. These domains are succeeded by a C-terminal domain (CTD, V^692^—R^776^). The domains are connected by linkers (L): LβD2-βD1 (L^35^—H^38^), LβD1-βD2 (P^323^—F^334^), and LβD2-CTD (G^680^—G^691^). The two propellers are offset from one another by a ~90° rotation about the intersection axis of the propellers’ planes. This causes the overall molecular structure to be reminiscent of an easy chair of approx. maximal dimensions 95 × 80 × 55 Å, with βD1 as the seat, βD2 the backrest, and CTD the backrest support (Fig. 5a). The two entry-side surfaces of the PorZ propellers mimic, respectively, the seating and reclining surfaces of the chair.

The seven blades of the propellers consist of a four-stranded (β1—β4) antiparallel β-sheet of simple up-and-down “W” connectivity or β-leaflet topology^40^. Two β1 strands and one β4 strand are interrupted by bulges (strands βD2-III-β1, βD2-IV-β1, and βD1-III-β4 Fig. 5b; for structural-element notation, see the legend to Fig. 5b). The blades are radially arranged around a central propeller shaft, which is lined by the respective first strands of each blade (β1) originating on the entry sides, and the strands of each leaflet are connected by short loops. Exceptions are those connecting βD2-I-β2 with βD2-I-β3, and βD2-IV-β2 with βD2-IV-β3, which span twelve and eight residues respectively, and protrude from the entry side of βD2 (Fig. 5a). Within each sheet and ongoing from β1 to β4, the strands accumulate a twist of ~45°, which is right-handed as usual for such structures^41^. Uniquely, blade IV of βD2 contains a metal, which was assigned to a calcium based on the octahedral ligand sphere, chemical nature of the six (oxygen) ligands, and binding distances of the ligands (~2.4 Å; see http://tanna.bch.ed.ac.uk/newtargs_06.html and ref. 42). The ion is bound by three main-chain oxygens, two aspartate side-chain oxygens, and a threonine side-chain oxygen (Fig. 5c). Each blade further consists of a C-terminal linker, which connects respective strand β4 with strand β1 of the downstream blade. Linkers vary in length between four and sixteen residues, and may contain extra regular secondary-structure elements such as β-ribbons (blades IV and V of βD2) and α-helices (blades I of βD2 and II of βD1; see Fig. 5b). The shafts of the PorZ propellers have an internal diameter spanning ~5–10 Å, and, in contrast to other propellers such as the four-fold hemopexin domains^38^, they do not show evidence of tight ion or ligand binding. We only found some loosely bound chemicals from the crystallization and vitrification conditions (data not shown), which could indicate potential binding sites of functional relevance. Further research will be required to verify this assertion, though.

In general, β-propeller symmetry ranges from four-fold to eight-fold, the most populated group being the seven-fold propellers^37^,^38^,^39^. This may result from the packing of the blades, which is considered more stable the larger the number of blades^39^. Thus, to circumvent low stability, four-fold and five-fold propellers incorporate additional elements to tether the circular arrangement, such as disulphide bonds between the N- and C-terminal blades^38^,^43^. Other mechanisms entail that the first blade is made up by the N-terminal part of the polypeptide chain for some of its strands and by the C-terminal part after completion of the entire propeller moiety for the remaining strands, thus featuring a kind of “velcro” mechanism^38^. In the case of PorZ, which consists of two consecutive seven-fold propellers, the N-terminal segment of the polypeptide chain follows such a mechanism and forms strand βD2-VII-β4, while the remaining three strands of this blade are provided by the polypeptide chain after forming both propellers (see Fig. 5b). After strand βD2-VII-β4, the polypeptide enters short, four-residue LβD2-βD1 and then forms βD1 starting with blade I. After βD1, which ends with strand βD1-VII-β4, LβD1-βD2 leads to strand βD2-I-β1. Altogether, βD1 and βD2 tightly approach each other through respective blades I and VII and linkers LβD2-βD1 and LβD1-βD2 (Fig. 5a). This may explain the stable, nearly perpendicular relative arrangement between propellers, which is reminiscent of that of the two four-fold propellers found in rabbit hemopexin^38^,^44^.

**Figure 6:**
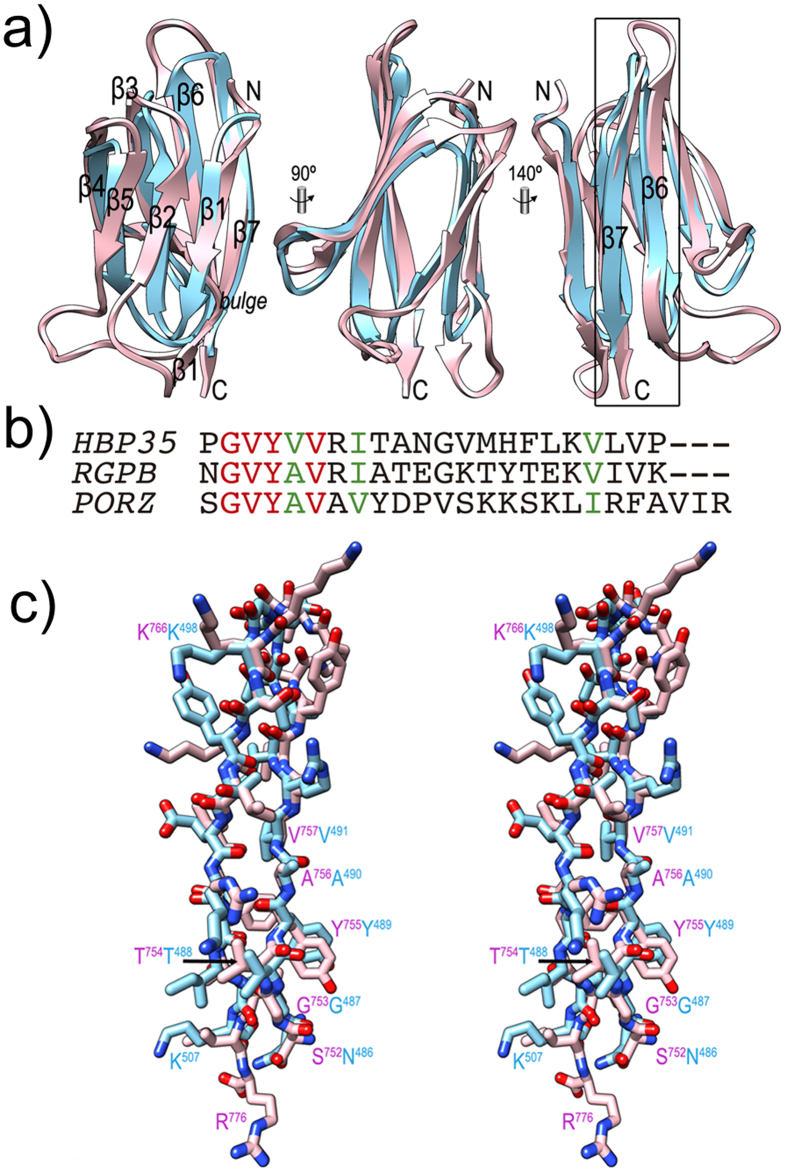
The C-terminal domain of PorZ. (**a**) Ribbon-plot of the CTDs of PorZ (pink) and RgpB (cyan) after optimal superposition facing the three-stranded front β-sheet (*left,* the seven constituting strands are labelled, see also Fig. 5b); after a vertical 90° rotation (*center*); and facing the four-stranded back β-sheet (*right*). C-terminal strands β6 and β7, which contain the reported molecular determinants for T9SS secretion^12^,^15^, are framed and labeled in the right panel. (**b**) Sequence alignment of the 22 C-terminal residues of HBP35 (UniProt Q8G962) and RgpB (UniProt P95493), and the 25 final residues of PorZ after the structural alignment of the structures of the latter two proteins (see also [**c**]). Identical residues are in red, similar ones in green. G-X-Y sequences are also found in PKD proteins within strands equivalent to CTD-β6. These are also seven-stranded immunoglobulin-like all-β domains^84^, although the function of the G-X-Y motif therein is unknown. In addition, the Y_Y_Y domain of BT4663 protein contains this signature^46^. (**c**) Detail in cross-eye stereo showing the strands framed in (**a**) as full-atom models, i.e. segments S^752^—R^776^ of PorZ (with pink carbons and magenta labels) and N^486^—K^507^ of RgpB (mature protein numbering as subscripts, add 229 for full-length protein numbering; cyan carbons and labels).

After βD2, LβD2-CTD leads to CTD (Figs 5a,b and 6a), which spans 85 residues and is organised as a β-sandwich consisting of a three-fold antiparallel β-sheet (strands CTD-β1, -β2, and -β5; CTD-β1 is interrupted by a bulge, see Fig. 5b) and a four-fold antiparallel β-sheet (CTD-β4, -β3, -β6, and -β7). Despite some general differences, in particular in loops, the architecture and strand connectivity is equivalent to that of the CTD of RgpB, which is the structural paradigm of a functional CTD required for T9SS secretion^12^. Both structures were superposed for 61 topologically equivalent residues, giving rise to an overall core *rmsd* of 1.8 Å and a sequence identity of 20% (see Fig. 6a–c). Although the latter value is rather low^45^, both structural and sequence similarity are particularly significant for the last ~25 residues of either molecule (Fig. 6b), a segment that encompasses the apparent signal recognized by T9SS for translocation^12^,^15^. A common pattern (G-V-Y-V/A-V-X-I/V) arises when the two sequences are aligned based on structural criteria (Fig. 6c) and the sequence of the T9SS cargo HBP35^15^ is further included (Fig. 6b). Thus, PorZ possesses a potentially functional CTD for T9SS secretion.

### Possible function of the PorZ propeller domains

The modular architecture of PorZ is reminiscent of that of the periplasmic sensor-domain moiety of protein BT4663 from *Bacteroides thetaiotaomicron,* which belongs to the human microbiome and is the most prevalent gut colonizer^10^. BT4663 is the transmembrane histidine kinase of a two-component signal transduction system engaged in detection and degradation of complex carbohydrates. BT4663 transits between distinct unbound and bound conformations, and in this way activates the intracellular kinase domain^46^. The general architecture of BT4663 is also found in related potential histidine kinases BT4673 and BT3049 from the same organism^47^. Similarly to PorZ, the three proteins likewise comprise two N-terminal seven-fold β-propeller domains and a C-terminal all-β domain, termed the Y_Y_Y domain for BT4663^46^.

However, while BT4663 and related proteins are dimeric^46^,^47^, PorZ was verified in size-exclusion chromatography to be a monomer in solution (data not shown). In addition, the relative arrangement of the three domains in either the bound or unbound forms, differs from that of PorZ. Differences also arise between the Y_Y_Y domain and PorZ CTD: although they share topology and strand connectivity, the former is much larger (120 *vs.* 85 residues) and has an extra strand. Moreover, while the Y_Y_Y domain functionally acts as a spacer from the cytoplasmic membrane surface further engaged in dimerization, PorZ CTD is a potential signalling domain (see below).

This notwithstanding, BT4663 may yet provide a hint to the molecular function of PorZ, as most of the known propeller domains coordinate ligands or catalyse reactions at or close to the central shafts on their entrance side^37^,^48^. Among its widespread functions are binding of sugar moieties and related molecules as reported for the five-fold propellers β-fructosidase, α-L-arabinanase and levan-sucrase. This is also the function of BT4663, which binds disaccharide ligands on the respective entry surfaces of both propeller domains, central to the shafts^46^. Along this line, the crystal structure of PorZ revealed potential polyethylene glycol fragments bound at the hinge between βD1 and βD2 on their entry side, as well as on the exit surface of βD2 (see Fig. 5a). Other smaller molecules were found in or on the shaft entrances (see above). Moreover, the two extended intra-leaflet loops, which protrude from the entry side of βD2 (see above), could also be potentially engaged in ligand binding, thus providing a functional explanation for their exceptional length. In any case, further experiments will be required to verify a potential glycan-binding function of PorZ as part of, or independently from, T9SS secretion.

### PorZ is itself secreted via T9SS with an intact CTD

**Figure 7:**
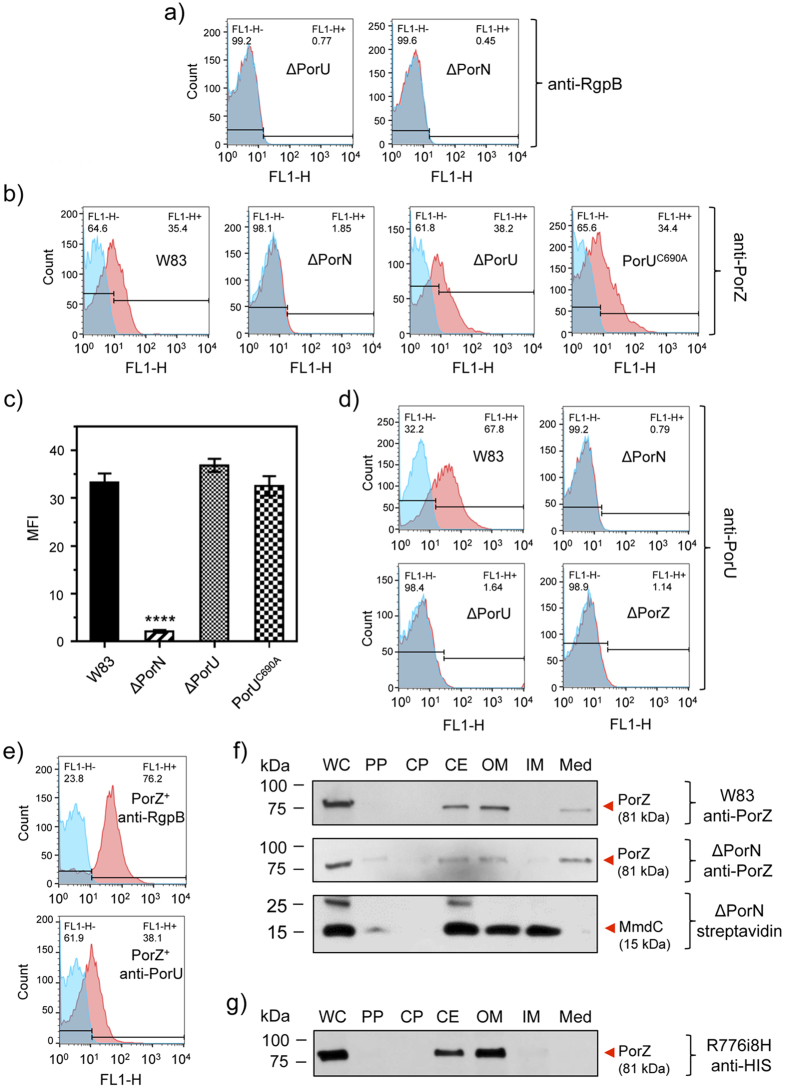
PorZ is secreted *via* T9SS with an intact CTD independently of PorU. Flow cytometry analysis using (**a**) anti-RgpB mAbs in ΔPorU and ΔPorN; (**b**) anti-PorZ antibodies in the wild type (W83), PorN-null mutant (ΔPorN), PorU-null mutant (ΔPorU), and PorU active-site inactivation mutant (PorU^C690A^); (**c**) PorZ surface exposure as mean fluorescent intensity (MFI) in different strains calculated from flow cytometry analysis (in duplicates) from three different cultures. Significant differences between the wild type and mutants were analyzed by one-way ANOVA with Bonferroni’s correction; ****P < 0.0001. Flow cytometry analysis using (**d**) anti-PorU antibodies in the wild type (W83), PorN-null mutant (ΔPorN), PorU-null mutant (ΔPorU), and ΔPorZ; (**e**) anti-RgpB and anti-PorU in *porZ* complemented strain (PorZ^+^). The result of using specific antibodies (red surface) and the negative isotype control (blue surface) are shown. (**f**) Western blot analysis of subcellular locations of PorZ in the ΔPorN mutant by probing with anti-PorZ antibodies as compared to the wild type (W83). Streptavidin conjugated to horseradish peroxidase was used to detect MmdC, a biotinylated IM-associated control protein. (**g**) Same as (**f**) but using anti-His antibodies to detect the CTD of PorZ in the strain expressing PorZ with an octahistidine tag at the C-terminus (R776i8H). Bacterial cultures were fractionated as described in the Methods section.

The presence of a CTD reminiscent of that found in T9SS cargos and components at the C-terminus of PorZ suggests that the protein is itself secreted to the surface by the same secretion system. In this event, PorZ would be absent from the bacterial surface in *P. gingivalis* mutants with dysfunctional T9SS, as shown for other T9SS cargos. To validate this hypothesis, we investigated the surface location of PorZ by flow cytometry analyses in three secretion-deficient mutants: ΔPorN, ΔPorU, and a mutant expressing inactive PorU, in which the catalytic cysteine had been mutated to alanine (PorU^C690A^). The defective secretion phenotype of the ΔPorN and ΔPorU mutants was confirmed by the lack of staining for RgpB (Fig. 7a). In contrast to the wild type, PorZ was absent from the cell surface of mutant ΔPorN but surprisingly not of mutants ΔPorU and PorU^C690A^ (Fig. 7b), which evinced similar levels to the wild type (Fig. 7c). This strongly argues that PorZ is translocated across the OM and anchored at the bacterial surface in a PorU-independent manner.

Due to the phenotypic similarity of the ΔPorZ mutant with the Skp-like PGN_0300 mutant, where PorU failed to insert into the OM^36^, we proceeded to investigate the surface exposure of PorU in the ΔPorZ and ΔPorN mutants as compared to wild type. Flow cytometry using an anti-PorU antibody revealed that PorU was absent from the cell surface of both ΔPorZ and ΔPorN, in stark contrast to the wild type (Fig. 7d). It was not present in the ΔPorU mutant as expected but the complementation of the ΔPorZ mutant restored the surface exposure of both PorU and RgpB (Fig. 7e). This result suggests that the export of PorU to the cell surface may be a complex process involving both an intact T9SS pathway as well as the Skp-like chaperone PGN_0300. It further suggests that PorU may be the last component of T9SS to be secreted to the surface as it requires a functional PorN and PorZ but not vice-versa, as PorZ was exported to the cell surface in the absence of wild-type or inactive PorU (Fig. 7a).

To determine the fate of PorZ in ΔPorN, we performed Western blot analysis on cultures that were quantitatively separated into whole cells and subcellular fractions. When compared with the wild type, the partition of PorZ between the OM and periplasmic fractions was only slightly higher in the periplasm of ΔPorN (Fig. 7f). This is in stark contrast with Rgps and Kgp, which are fully processed and associated with the OM in the wild type (Fig. 2a,b) but found in unprocessed and partially processed forms in the periplasm of ΔPorN (Supplementary Fig. S5). The same distribution of unprocessed gingipains in the periplasm is apparently a hallmark of all T9SS secretion mutants characterized to date, including ΔPorZ (Fig. 2), PorT^49^, PorU^17^, Sov^50^, and LptO^51^. Taken together, our results unambiguously show that PorZ is associated with the OM, and its surface exposure is dependent on PorN but not PorU (Fig. 7b,c). Conversely, PorU exposure on the cell surface requires the presence of PorZ (Fig. 7d,e).

**Figure 8:**
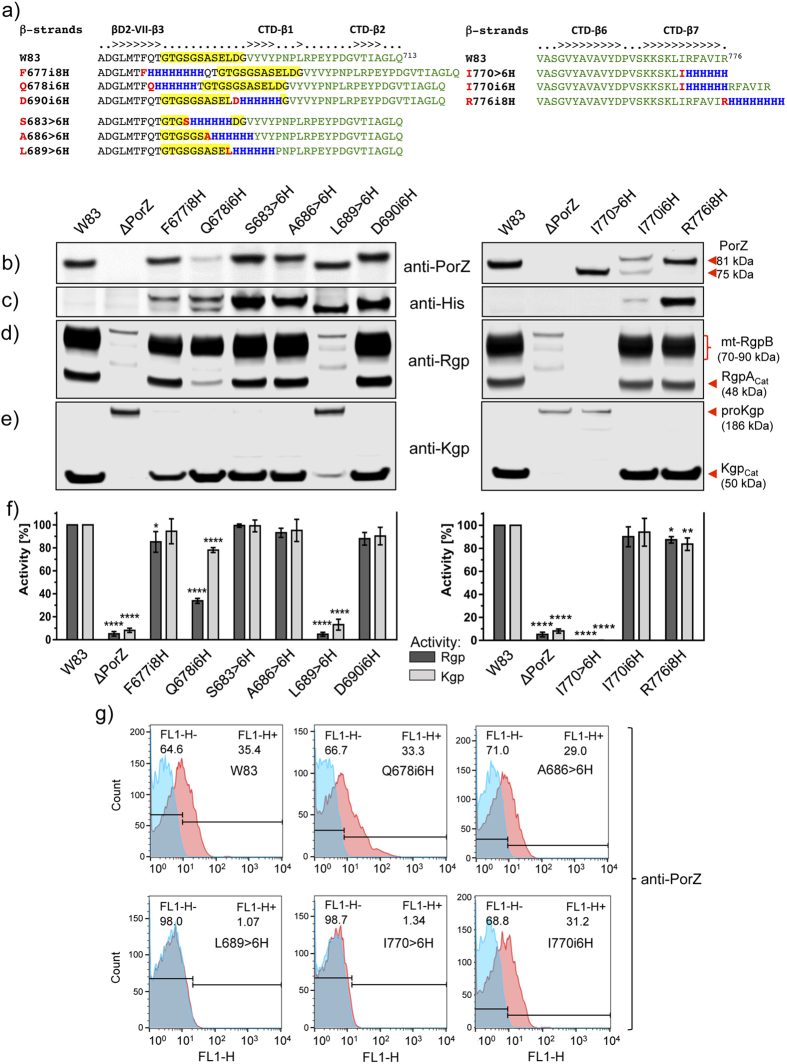
Effects of the introduction of, or replacement with, oligohistidines on PorZ function. (**a**) Location of insertions (i) and substitutions (>) of consecutive residues by polyhistidines at the junction (residues G^680^-G^691^, over yellow background) between CTD (green font) and the preceding domain of PorZ (F677i8H, Q678i6H, S683 > 6 H, A686 > 6 H, L689 > 6 H and D690i6H) or at the C-terminus (I770 > 6 H, I770i6H, and R776i8H). β-strands are indicated above the alignment. (**b**–**e**) Wild-type and mutant strains were grown to OD_600_ = 1.0 and whole cultures were subjected to Western blot analysis with anti-PorZ (**b**), anti-polyhistidine (**c**), anti-Rgp (**d**) and anti-Kgp (**e**) antibodies. (**f**) The same strains were used for gingipain activity assays. (**g**) The level of surface exposure of PorZ in various mutants was analyzed by flow cytometry using anti-PorZ antibodies (red) and negative isotype control (blue). Representative histograms are shown from three independent experiments.

As to which variant of PorZ is found on the cell surface, the relative molecular mass of a PorZ-immunoreactive band in SDS-PAGE was ~80 kDa, which suggests that the protein is full length, without the signal peptide (theoretic molecular mass: 81 kDa). To verify this contention, we constructed *P. gingivalis* mutant strain R776i8H, which expresses PorZ with an octahistidine at the C-terminus (see also the next section). This mutant possesses a secretory phenotype that is indistinguishable from the wild type, as determined by colony pigmentation (data not shown), cellular distribution (Fig. 7e) and gingipain activity (Fig. 8f). Western blot analysis with anti-His-tag antibodies revealed reactivity to a band of ~80 kDa, which confirmed the presence of intact CTD in the mature PorZ protein (Fig. 7g). This observation is consistent with proteomics data reporting that PorZ appears to retain its CTD and does not undergo A-LPS modification as seen in other T9SS cargos^27^.

### An intact CTD is required for cargo translocation across the OM and post translational processing

It was previously described that an intact CTD was needed for proper secretion, because its removal or C-terminal truncation prevented OM translocation and resulted in accumulation of the cargo protein in the periplasm^32^. However, insertion of additional residues into the linker region between the CTD and the preceding immunoglobulin superfamily domain of RgpB resulted in cleavage of the CTD but prevented A-LPS attachment. In addition, the N-terminal pro-domain responsible for latency maintenance in pro-RgpB^52^ was cleaved off, so soluble, fully active gingipain was secreted into the medium^53^.

The crystal structure of PorZ revealed that CTD is preceded by domain βD2, with the inter-domain linker LβD2-CTD spanning residues G^680^-G^691^ (see above). To study the effect of oligohistidine insertions or replacements on PorZ expression, processing and translocation, we inserted hexa/octahistidines or replaced six consecutive residues with histidines within LβD2-CTD (six mutants) and at the C-terminus of the CTD (three mutants) (Fig. 8a). Western blot analysis with either anti-PorZ (Fig. 8b) or anti-His-tag antibodies (Fig. 8c) revealed ~81-kDa (full-length) and/or ~75-kDa (truncated) immunoreactive bands, which were found with the former antibody in all strains tested except ΔPorZ (Fig. 8b). Both forms were found in mutant I770i6H with the anti-PorZ antibody, and the shorter fragment was exclusive for the I770 > 6 H and L689 > 6 H mutants. In turn, results with anti-His-tag antibodies showed the oligohistidine motif in full-length and/or truncated PorZ in all mutants expressing the protein tagged in the LβD2-CTD segment (Fig. 8c, *left panel*). In the case of the C-terminal mutants, the tag was detected only in two mutants expressing, respectively, PorZ C-terminally extended with an octahistidine attached to C-terminal residue R^776^ (mutant R776i8H) and when hexahistidine was inserted after I^770^ (mutant I770i6H) (Fig. 8c, *right panel*). In contrast, no tag was detected in the ~75-kDa form of PorZ with hexahistidine substituting the six most C-terminal residues (I770 > 6 H) and when inserted in the middle of the last β-strand (CTD-β7) of the protein (I770i6H). The lack of reactivity of the ~75-kDa band with anti-His-tag antibodies in this mutant clearly indicates that mutated PorZ was cleaved at the C-terminus, losing ~5-kDa.

To verify on which side (N- or C-terminal) the protein was cleaved in LβD2-CTD linker mutants, we purified the truncated PorZ variant derived from the L689 > 6 H mutant by affinity chromatography, and subjected the protein to N-terminal sequencing analysis. The N-terminus was found to be intact, so cleavage must have occurred at the C-terminus, downstream of the inserted histidine-tag. The same can be assumed for the ~75-kDa PorZ variant derived from mutant Q678i6H.

Next, we determined the function of the PorZ variants in the processing and activation of gingipains. A membrane-bound form of RgpB (mt-RgpB) and the mature RgpA catalytic domain (RgpA_cat_) were clearly visible in the wild type and in mutants F677i8H, Q678i6H, S683 > 6 H, A686 > 6 H, D690i6H, I770i6H and R776i8H (Fig. 8d), which all possessed full-length PorZ (Fig. 8b,c). Conversely, mutants L689 > 6 H and I770 > 6 H, which do not have this PorZ variant, entirely lacked both mt-RgpB and RgpA_cat_ (Fig. 8d). Similarly, the mature catalytic domain of Kgp was found only in mutants possessing full-length PorZ, while only the Kgp precursor was present in mutants missing full-length PorZ (Fig. 8e). Lack of gingipain processing in these mutants correlated with substantially lower Rgp and Kgp activities in the culture than in the wild type (Fig. 8f). The only exception was mutant Q678i6H, which seemed to process gingipains, but with much lower yields than the wild type (Fig. 8f). Finally, flow cytometry studies (Fig. 8g) revealed that all mutants with full-length PorZ displayed surface-exposed PorZ at levels comparable to those of the wild type. Conversely, strains with truncated PorZ (L689 > H6 and I770 > 6 H) had negligible amounts of this protein on the cell surface, despite comparable expression levels of modified PorZ, as shown by Western blot (Fig. 8b,g).

## Conclusions

Members of the dysbiotic oral microbiome, such as *T. forsythia* and *P. gingivalis*, and some environmental Gram-negative bacteria uniquely possess T9SS dedicated to the export of proteins. This multi-component machinery consists of a minimum of twelve Por proteins and is responsible for the secretion of at least 32 proteins in *P. gingivalis*. The export signal is not a flexible peptide but rather a full ~70-residue protein domain located at the C-terminus called the CTD, which is removed upon secretion. Here, we discovered a new component of T9SS, PorZ, and found it loosely associated with the *P. gingivalis* cell surface in a manner independent of A-LPS anchorage. In T9SS cargo enzymes such as PPAD and gingipains, its absence prevented proper secretion to the extracellular medium, cleavage of CTDs, activation from precursor forms, and anchoring in the OM. In this case, cargos accumulated in the periplasmic space and the culture medium, the latter owing to leaky OM architecture. Increased mRNA expression of CTD-cargo proteins in the ΔPorZ mutant suggested that absence of these functional proteins on the cell surface induced a feedback response, which augmented expression of these proteins and of some T9SS components, presumably in an effort to secrete more cargo proteins to the surface.

To gain insight into the molecular determinants of PorZ function, we solved its full-length crystal structure, which revealed two N-terminal seven-fold β-propeller domains in tandem. Such domains are widely used in macromolecular recognition and are engaged in protein-protein and protein-substrate interactions, as the architecture is generally versatile enough to enable binding of small molecules like sugars^43^. Based on structural similarities with sugar-binding proteins from *B. thetaiotaomicron* from the human gut microbiome, we hypothesized that PorZ may likewise have a potential glycan-binding function as part of, or independently from, T9SS secretion. Downstream of the β-propeller domains, PorZ further comprises a C-terminal seven-stranded β-sandwich, which conforms to the canonical CTDs of other T9SS-secreted proteins. This allowed us to further hypothesize that PorZ may itself be a T9SS cargo. Further functional studies showed that PorZ is actually transported to the cell surface *via* T9SS as a full-length protein with an intact CTD- a translocation that was independent of the presence or activity of sortase PorU. Consistently, PorZ was absent from the surface but apparently remained associated with the periplasmic side of the OM in a T9SS mutant lacking protein PorN.

We further studied the effect of oligohistidine insertions or replacements on PorZ expression, processing and translocation to the bacterial surface, and inserted hexa- or octahistidines or replaced six consecutive residues with histidines within linker LβD2-CTD and at the C-terminus of CTD. Collectively, these mutations revealed that although CTD can be elongated at the C-terminus without affecting the secretory phenotype of the mutant, alteration of the native C-terminal sequence was absolutely prohibited^26^,^53^. Interestingly, in both cases truncation/substitution of C-terminal residues resulted in partial cleavage of CTD and accumulation of modified protein in the periplasm. However, modifications of the loop preceding CTD in PorZ had a negligible effect on protein function, as long as they did not affect strand CTD-β1. In T9SS cargos, such mutations decouple CTD removal by sortase PorU from the attachment of A-LPS, thus releasing fully-processed cargo proteins into the culture medium. This accounts for different functions of the linker domain preceding CTD, which is unstructured in cargos and provides a cleavage/A-LPS attachment site for sortase PorU^12^. In stark contrast, the intra-domain loop is well-structured in PorZ and resistant to proteolysis by PorU, thus suggesting an important role of CTD in PorZ function in T9SS.

To sum up, the reported full molecular and functional characterization of PorZ, a novel essential surface component of T9SS, contributes to our understanding of protein secretion as part of host-microbiome interactions by dysbiotic members of the human oral cavity.

## Materials and Methods

### Bacterial strains and general growth conditions

*Porphyromonas gingivalis* strain W83 (wild type and mutants, listed in Table 1) was grown in enriched tryptic soy broth (eTSB per liter: 30 g trypticase soy broth, 5 g yeast extract, 5 mg hemin, pH 7.5; further supplemented with 0.5 g L-cysteine and 2 mg menadione) or on eTSB blood agar (eTSB medium *plus* 1.5% agar, further supplemented with 4% defibrinated sheep blood) at 37 °C in an anaerobic chamber (Don Whitley Scientific, UK) with an atmosphere of 90% nitrogen, 5% carbon dioxide and 5% hydrogen. *Escherichia coli* strains (listed in Table 2), used for all plasmid manipulations, were grown in Luria-Bertani (LB) medium and on 1.5% agar LB plates. For antibiotic selection in *E. coli*, ampicillin was used at 100 μg/ml, kanamycin at 50 μg/ml, spectinomycin at 50 μg/ml and erythromycin at 250 μg/ml. *P. gingivalis* mutants were grown in the presence of erythromycin at 5 μg/ml and/or tetracycline at 1 μg/ml.

### Generation of *P. gingivalis* ΔPorZ, ΔPorU and ΔPorN deletion mutants

All *P. gingivalis* deletion mutants were generated by homologous recombination, as previously described for other genes^54^. Three suicide plasmids were generated analogously. For deletion mutant ΔPorZ, briefly, two 1-kb flanking regions on either side of the *porZ* gene were amplified from genomic DNA by PCR with primer pairs PG1604FrANdeIF/PG1604FrASmaR (for primers and sequences, see Supplementary Table S2) and PG1604FrBXbaIF/PG1604FrBSalIR. The PCR product was cloned into plasmid pUC19 with an inserted erythromycin-resistance (*erm*) cassette (*ermF-ermAM*), amplified from plasmid pVA2198^55^ with primers ermFAMSmaIF/ermFAMSalIR. Correct placement and orientation of the DNA segments in resulting plasmid p1604AeB-D was confirmed by sequencing.

A plasmid for mutant ΔPorN, designated p291AeB-C, was generated similarly using vector pUC19 and primer pairs PG291FrBXbaIF/PG291FrBPstIR and PG291FrANdeIF/PG291FrASmaIR, as well as primers ermFAMSmaIF/ermFAMXbaIR to introduce cassette *ermF-ermAM* between chromosomal DNA fragments.

In like manner, a plasmid for mutant ΔPorU was obtained using primer pairs PorUFrag5_F/PorUFrag5_R and PorUFrag3_F/PorUFrag3_R to amplify 1-kb upstream and downstream flanking regions of the *porU* gene respectively. Blunt-end PCR products were cloned into plasmid pCR-BluntII-TOPO using the Zero Blunt TOPO PCR cloning kit (Invitrogen) and then subcloned into a pUC19 plasmid containing a promoterless *erm* resistance gene^55^ yielding plasmid pPorU/pUC19/Erm.

The resulting suicide plasmids for generating the ΔPorU, ΔPorZ and ΔPorN mutants were introduced by electroporation into electrocompetent wild-type *P. gingivalis* cells prepared as previously described^56^. Resultant clones were selected on erythromycin plates and double-crossover genomic recombination was confirmed by DNA sequencing of the manipulated region. Isogenic mutant ΔPorU was further verified for inoffensiveness on translation of the downstream gene encoding protein LptO (*alias* PorV and PG0027) through Western blot analysis using rabbit polyclonal anti-LptO antibodies.

### Generation of *P. gingivalis* PorZ and PorU mutants

Master plasmid p1604CeB-H was created for *porZ* modifications in a pUC19 background through sequential insertion of the following PCR fragments: a 2.3-kb PCR fragment encoding most of the *porZ* gene (primer pair: PG1604FrANdeIF2/PG1604FrCSmaR); an *ermF-ermAM* cassette (primer pair: ermFAMSmaIF/ermFAMSalIR); and a 1-kb downstream fragment of the *porZ* gene (primer pair: PG1604FrBXbaIF/PG1604FrBSalIR). The same method was used to create pPorU-E master plasmid. Here, PCR fragments consisted of a 2.5-kb fragment partially encompassing the *porU* gene (primer pair: Pg26_AR/Pg26_AF); an *ermF-ermAM* cassette (primer pair: Pg26_ER/Pg26_EF); and a 0.9-kb downstream fragment of the *porU* gene (primer pair: Pg26_BR/Pg26_BF).

Similarly to the reported generation of RgpB and Cpg mutants^53^, SLIM mutagenesis^57^ was employed to replace the catalytic cysteine of PorU (C690) with alanine within the pPorU-E master plasmid to yield mutant PorU^C690A^.

PorZ mutants including insertion of an oligohistidine-tag or substitution of six consecutive residues with six histidines at various locations at the inter-domain linker between domains βD2 and the CTD (mutants PorZ F677i8H, Q678i6H, S683 > 6 H, A686 > 6 H, L689 > 6 H, and D690i6H) or at the C-terminus (mutants PorZ I770 > 6 H, I770i6H, and R776i8H) were generated by the SLIM method within the *porZ* master plasmid (see Supplementary Table S2). Mutated plasmids were verified by DNA sequencing of the pertinent region before being electroporated into wild-type *P. gingivalis* for homologous recombination^54^. Resistant clones were selected using erythromycin-selective media.

### Construction of *porZ* complementation strain (PorZ^+^)

To construct a complementation plasmid (pT-COW-porZ), a 3166-bp fragment containing the entire *porZ* coding sequence *plus* 544 bp upstream and 291 bp downstream was obtained by PCR from *P. gingivalis* genomic DNA with primers pT-COWporZForSalI and pT-COWporZRevNheI. Next, the fragment was cloned into SalI and NheI restriction sites of the pT-COW plasmid^58^ by a standard procedure. After pT-COW-porZ sequencing, the plasmid was introduced into ΔPorZ strain by conjugation according to^59^.

### Culture partitioning and subcellular fractionation

Two-day-old cultures in the stationary phase of wild-type and mutant *P. gingivalis* were adjusted to OD_600_ = 1.5 and designated “whole culture” (WC). Bacterial cells were collected from WC by centrifugation (8000× *g*, 15 min). The cell pellet was then washed and resuspended in PBS up to the initial volume of WC, and centrifuged. This fraction is referred to as “washed cells”. The collected cell-free culture medium was ultracentrifuged (100,000× *g*, 1 h) to remove vesicles and the supernatant was concentrated 10-fold by ultrafiltration with filter devices of 10-kDa cut-off; this fraction was designated “medium” (Med). Subcellular fractionation was carried out as described^60^, with some modifications. Briefly, bacteria were cultured until OD_600_ = 1.2 and fraction “cell extract” was obtained by centrifugation of 0.5 ml of WC and a single wash step with PBS. The rest of the culture (7.5 ml) was centrifuged to produce a supernatant and a pellet. The supernatant was filtered through a 0.22-μM syringe filter and concentrated three times with centrifugal filter devices of 10-kDa cut-off to obtain the “medium” fraction. The pellet was rinsed with PBS and suspended in 2.5 ml buffer containing 0.25 M sucrose, 30 mM Tris·HCl, pH 7.6. After a 10-min incubation period, cells were pelleted (12,500× *g*, 15 min) and rapidly resuspended in 2.5 ml of cold distilled water to disrupt outer membranes. After a further 10-min incubation period, spheroplasts were obtained and separated by centrifugation (12,500× *g*, 15 min). The supernatant was collected and designated as the “periplasmic” fraction. Next, spheroplasts were washed once with PBS, suspended in 2.5 ml of fresh PBS, and disrupted by brief sonication. The resulting solution was ultracentrifuged (150,000× *g*, 1 h) and the soluble fraction was designated as the “cytoplasmic” fraction. The pellet was resuspended in 2.5 ml PBS and briefly sonicated, and was designated as the “cell envelope” fraction. Inner membranes were solubilized by incubation in PBS supplemented with 1% Triton X and 20 mM magnesium chloride (final concentration) for 20 min. Membranes were separated by ultracentrifugation (150,000× *g*, 1 h) and the resulting supernatant was dubbed the “inner membrane” fraction. The pellet was then resuspended in PBS and sonicated, which resulted in the “outer membrane” fraction. All fractions were supplemented with peptidase inhibitors (5 mM tosyl-L-lysyl-chloromethane hydrochloride [TLCK], 1 mM 2,2’-dithiodipyridine [DTDP], 1x EDTA-free protein inhibitor cocktail (all from Roche) before storage at −20 °C.

### Antibodies

Rabbit anti-RgpB antibodies, which also recognize the catalytic domain of RgpA^61^, were used as the primary antibody. Mouse monoclonal antibodies specific for the Kgp catalytic domain and a single epitope on RgpB (18E6), respectively, were developed at the University of Georgia (GA, USA) Monoclonal Antibody Facility using recombinant protein as the antigen^54^,^62^. A rabbit polyclonal antibody recognizing *P. gingivalis* peptidylarginine deiminase (PPAD) was generated by Cambridge Research Biochemicals, Billingham, UK^63^. Mouse monoclonal antibodies specific for PorU were developed using standard procedures in the Laboratory of Monoclonal Antibodies at the Malopolska Center of Biotechnology, Jagiellonian University (Krakow, Poland) with recombinant PorU as the immunizing antigen.

Mouse polyclonal antibodies against protein PorZ were obtained according to standard procedures. Briefly, six-week old Balb/C mice were injected intraperitoneally with 100 μg of antigen diluted in PBS and mixed 1:1 with Complete Freund’s Adjuvant (Sigma Aldrich). Subsequent immunizations were performed with 50 μg of antigen mixed with Incomplete Freund’s Adjuvant (Sigma Aldrich). The serum titer of polyclonal antibodies specific to the antigen was determined by ELISA according to standard procedures. After administration of *an adequate anaesthetic* (ketamine and xylazine), blood was collected from immunized animals by cardiac puncture. Polyclonal antibodies were purified from the serum by affinity chromatography with protein G, according to the manufacturer’s instructions (Thermo Fisher Scientific), and then dialyzed against sterile PBS. Finally, the antibodies were purified by affinity chromatography employing recombinant PorZ, which was covalently bound to Pierce™ NHS-Activated Agarose Spin Columns (Thermo Fisher Scientific), according to the manufacturer’s instructions. This work was carried out in the Laboratory of Monoclonal Antibodies at the Małopolska Center of Biotechnology, Jagiellonian University (Krakow, Poland). Mouse monoclonal antibodies against PorZ were also generated commercially by Abmart (Shanghai, China) using a synthetic peptide of sequence E-K-G-R-K-T-T-Q-F-P. All experiments were performed in accordance with relevant guidelines and regulations.

### Western blot analysis

Sample preparation, separation by SDS-PAGE, electro-blotting onto polyvinylidene difluoride or nitrocellulose membranes and blocking were performed as described previously^53^. RgpA and RgpB catalytic domains were detected with rabbit polyclonal anti-Rgp antibodies; Kgp with mouse monoclonal anti-Kgp antibodies; PPAD with rabbit polyclonal anti-PPAD antibodies; and PorZ with mouse polyclonal anti-PorZ antibodies (see also above). In addition, oligohistidine-tagged PorZ was detected using mouse monoclonal THE^TM^ His-Tag antibodies (Genscript, USA) at 0.125 μg/ml. Development with polyclonal anti-mouse or anti-rabbit horseradish peroxidase-conjugated secondary antibody (BD Pharmingen and Amersham Pharmacia) was carried out using the ECL Western Blotting substrate kit according to the manufacturer’s instructions (Pierce, UK). Streptavidin conjugated to horseradish peroxidase was used to detect MmdC, a biotinylated IM-associated protein.

### Dot blot analysis

*P. gingivalis* cells were harvested at OD_600_ = 1.5, washed and resuspended in cold PBS to yield OD_600_ = 1.0. Half of the suspension was sonicated to disrupt cell membranes, and 5 μl of either intact or disrupted cell suspension were spotted onto 0.22-μm nitrocellulose membranes and air dried. Subsequent steps were performed as described for Western blot analysis.

### Flow cytometry analysis

Wild-type and mutant *P. gingivalis* strains were grown in eTSB until they reached the late exponential or early stationary growth phase (OD_600_~1.2–1.5). Bacterial cells were harvested by centrifugation, washed twice with PBS and adjusted to OD_600_ = 1.0 with buffer (PBS supplemented with 1% bovine serum albumin). Then, 100 μl of cell suspension was transferred to a 96-well plate and incubated for 30 min with the previous buffer. Cells were collected by centrifugation (5,000× *g*, 5 min) and the pellet was resuspended in the previous buffer containing mouse antiserum specific for PorZ, RgpB or PorU at a total protein concentration of 30 μg/ml, and incubated for 30 min. Thereafter, cells were centrifuged (5,000× *g*, 5 min) and the newly obtained pellet was resuspended in the previous buffer containing goat anti-mouse antibody conjugated with fluorescein isothiocyanate (Abcam) at 1:200 dilution and incubated for 30 min. Cells were washed twice with PBS after each incubation with antibodies or streptavidin-Alexa Fluor 488 conjugate. The whole staining procedure was performed on ice. After staining, one-color flow cytometry analyses were performed using a FACSCalibur apparatus (BD Biosciences) operating with CellQuest software (BD Biosciences). Graphs were prepared using the *FLOWJO v.10 program* (Ashland, USA).

### Electron microscopy

Wild-type *P. gingivalis* cells grown in eTSB were harvested at 4,000× *g*, washed once with PBS, and then fixed with 2% [w:v] paraformaldehyde, 0.02% [v:v] glutaraldehyde fixative for 30 min. Cells were washed twice with PBS, then incubated with glycine (2 mg/ml) for 10 min. After a further wash with PBS, cells were dehydrated through graded alcohol, embedded in London Resin White (London Resin, UK) in gelatin capsules, and polymerized at 55 °C for 20 hours. Samples were then sectioned to ultra-thin sections (70 μM) on a Leica UC6 Ultramicrotome and picked up on Nickel 200 Square mesh fine bar grids (Gilder grids, UK) coated with 0.12% Formvar and 0.12% Pioloform. Sections were subsequently blocked with 10% [v:v] goat blocking buffer (5% bovine serum albumin [BSA], 10% normal goat serum, 0.2% cold water fish-skin gelatin, 10 mM sodium azide, pH 7.4) for 1 h, washed six times with incubation buffer (0.2% Aurion BSA-c, 10 mM sodium azide), and then incubated with monoclonal anti-PorZ antibodies (5 μg/ml) for 1 h. Sections were then washed six times with incubation buffer and then incubated with goat anti-mouse-5-nm gold conjugate for 1 h (BBI Solutions 1/40). The sections were then washed six times with PBS, post-fixed with 2% [v:v] glutaraldehyde for 5 min, and finally washed twice with PBS and six times with water before drying.

Grids were then treated with 2% osmiate vapour for 5 min, stained first with 1% uranyl acetate and then with Reynolds’ lead citrate^64^. Grids were visualized in a 100-kV FEI CM120 electron transmission microscope and digital images were taken with a SIS Morada CCD camera.

### Quantitative RT-PCR analysis

Quantitative RT-PCR data were obtained from four separate experiments. Briefly, wild-type and ∆PorZ cells were cultured to the mid-exponential phase and collected at OD_600_ = 0.8–0.9. Cells were stabilized with RNAprotect Bacteria Reagent (Qiagen, Germany) prior to mRNA extraction with the RNAqueous Total RNA Isolation Kit (Ambion, USA). Reverse transcription was carried out on 2 μl of total RNA using the AffinityScript qPCR cDNA Synthesis Kit (Stratagene, Australia) in 10 μl total volume, according to the manufacturer’s instructions. Quantitative RT-PCR was carried out in 25-μl singleplex reactions using 5 μl of a 1:50 dilution of cDNA along with TaqMan probe and primers against genes related to T9SS components (*porT*, *sov*, *porU*, *lptO*, *porN*, *porO* and *porW*) or cargos (*rgpB*, *kgp*, and *cpg70*). A housekeeping DNA gyrase (*gyrA*) gene and ribosomal 16 s (*r16s*) were used as calibrator genes (see primers and probes in Supplementary Table S2). Thereafter, thermal cycling with a Stratagene Mx3005 P Real-Time PCR System^®^ using the Brilliant^®^ II QPCR Master Mix (Stratagene, Australia) entailed activation for 15 min at 95 °C *plus* 40 cycles of annealing at 95 °C for 20 s and extension at 60 °C for 1 min. Fluorescence intensities were normalized against a passive fluorophore carboxy-X-rhodamine (ROX) present in the Master Mix and converted to absolute quantities using standard curves. The expression of target genes was normalized to the housekeeping *gyrA* and *r16s* genes independently by expressing the data as the average threshold cycle value of the target gene divided by that of either housekeeping gene. The average ratio in the wild type was arbitrarily set to 1.0 for reference.

### Large-scale expression, purification and oligomeric characterization of recombinant PorZ

Genomic DNA was isolated from wild-type *P. gingivalis* strain W83 using the Genomic Mini System (A&A Biotechnology, Gdansk, Poland), according to the manufacturer’s instructions. The gene encoding residues Q^26^-R^776^ (PorZ residue numbers in superscript notation, see UniProt entry Q9S3Q8), i.e. without the predicted signal peptide (M^1^-A^25^), was amplified by PCR, purified, and cloned into the pGEX-6P-1 expression vector using SmaI/NotI sites (for primer sequences, see Supplementary Table S2). The resulting recombinant product encoded an N-terminal glutathione-S-transferase (GST) moiety, a PreScission protease cleavage site, and the cloned protein. The plasmid was verified by DNA sequencing and transformed into *E. coli* expression strain BL21 (DE3) under the control of the T7 promoter. Transformed cells were grown in LB medium at 37 °C until OD_600_ = 0.75–1.0, and then for a further 30 min at 20 °C. Protein expression was induced by the addition of 0.25 mM isopropyl-1-thio-β-D-galactopyranoside and allowed to proceed for 8 h at 20 °C. Thereafter, cells were harvested by centrifugation (15 min, 6,000× *g*, 4 °C), re-suspended in PBS supplemented with 0.02% sodium azide (15 ml per pellet from 1 liter of culture), and subsequently lyzed by sonication. Cell lysates were clarified by centrifugation (50 min, 50,000× *g*, 4 °C), filtered through a 0.45-μm syringe filter, and loaded onto a glutathione-Sepharose 4 Fast Flow column equilibrated with PBS supplemented with 0.02% sodium azide at 4 °C. The GST-tag was removed from the recombinant protein bound to the column by intra-column cleavage with PreScission Protease (Amersham Biosciences). The cloning strategy left ten residues (G-P-L-G-S-P-E-F-P-G; confirmed by N-terminal sequencing) attached to the N-terminus of the recombinant protein. The latter was subsequently purified by size-exclusion chromatography using a HiLoad 16/60 Superdex 200 pg (GE Healthcare LifeSciences) column. A selenomethione variant of PorZ was obtained in the same way except that selenomethionine was used instead of methionine in cell cultures. Size-exclusion chromatography in a calibrated Superdex 200 column further revealed that purified recombinant PorZ eluted as a monomer (Supplementary Fig. S4).

### Purification of histidine-tagged PorZ from *P. gingivalis* mutant

Among the aforementioned PorZ histidine-insertion mutants (see section *Generation of P. gingivalis PorZ and PorU mutants*), mutant PorZ L689 > 6 H yielded PorZ protein truncated by ~5 kDa. To determine the cleavage site, the protein was purified for N-terminal sequence analysis. Briefly, the mutant cell culture supernatant was clarified by centrifugation (8,000× *g*, 30 min, 4 °C). The pellet was resuspended in cold PBS, and peptidase inhibitors TLCK (final concentration 5 mM) and DTDP (1 mM) were added, in addition to 2% protein inhibitor cocktail (Sigma). The sample was then sonicated and ultracentrifuged (150,000× *g*, 1 h, 4 °C), and the clarified lysate was dialyzed against buffer (20 mM sodium phosphate, 500 mM sodium chloride, 20 mM imidazole, 0.02% sodium azide, pH 7.4), concentrated by ultrafiltration in a filter device with 10-kDa cut-off, and stirred gently overnight with Nickel Sepharose 6 Fast Flow resin (GE Healthcare), previously equilibrated with the above buffer. The resin was then loaded on a column, washed until OD_280_ reached background level, and eluted with 20 mM sodium phosphate, 0.5 M sodium chloride, 500 mM imidazole, pH 7.4 to recover mutant protein PorZ L689 > 6 H. Fractions containing the highest protein concentration - as determined by a bicinchoninic acid assay - were pooled, concentrated, and further analyzed. Purified protein was resolved by SDS-PAGE, transferred onto a polyvinylidene difluoride membrane, and stained with Coomassie Brilliant Blue. The band of protein was excised and subjected to N-terminal sequencing.

### Mass spectrometry of media samples

Proteins in concentrated (20x) particle-free growth medium were ultracentrifuged and resolved by SDS-PAGE (NuPAGE Novex Bis-Tris System). Gels were stained with SimplyBlue™SafeStain (Novex) and washed in distilled water. Gel lanes were cut into sections and subjected to mass spectroscopy and follow up analysis, as described earlier^65^.

### Enzyme activity assays

The extracellular hydrolytic activity of *P. gingivalis* proteases was determined as described previously^66^,^67^. Briefly, the chromogenic *p*-nitroanilide (*p*NA) substrates benzoyl-Arg-*p*NA, acetyl-Lys-*p*NA, N-Gly-Pro-*p*NA and N-Ala-Phe-Pro-*p*NA (all from Bachem) were used to detect RgpA/B, Kgp, dipeptidyl peptidase IV, and prolyl tripeptidyl peptidase A, respectively. Samples were preincubated in buffer in 96-well plates prior to the addition of substrate to a total volume of 200 μl. The final concentration of each substrate was 1 mM. The rate of substrate hydrolysis, monitored through accumulation of *p*NA, was followed at 405 nm and the activity of each enzyme was given as mOD/min/μl or as a % of wild-type activity.

### Crystallization and diffraction data collection

Crystallization assays were performed by the sitting-drop vapor diffusion method. Reservoir solutions were prepared by a Tecan robot and 100 nl crystallization drops were dispensed on 96 × 2-well MRC plates (Innovadyne) by a Phoenix nanodrop robot (Art Robbins) or a Cartesian Microsys 4000 XL robot (Genomic Solutions) at the joint IBMB/IRB Automated Crystallography Platform (www.sbu.csic.es/facilities/automated-crystallographic-plattform). Plates were stored in Bruker steady-temperature crystal farms at 4 °C or 20 °C. Successful conditions were scaled up to the microliter range in 24-well Cryschem crystallization dishes (Hampton Research). The best crystals of PorZ were obtained at 20 °C in drops containing 2 μl of protein solution (at 6.5 mg/mL in 5 mM Tris·HCl, pH 8.0), 1 μL of reservoir solution (8% polyethylene glycol 10,000, 0.2 M zinc acetate, 0.1 M sodium cacodylate, pH 6.5) and 0.25 μl of additive solution (0.1 M calcium chloride). Selenomethionine-containing crystals were obtained similarly with 1 μl of the same reservoir solution, 1 μl of protein solution (at 5 mg/mL in 5 mM Tris·HCl, pH 8.0) and 0.35 μl of additive solution (7% butanol). Crystals were cryo-protected by rapid passage through drops containing increasing amounts of glycerol (up to 30% [v/v]). A complete dataset was collected at 100 K from a liquid-N_2_ flash cryo-cooled (Oxford Cryosystems 700 series cryostream) native crystal at beam line ID23–2 of ESRF synchrotron (Grenoble, France) using a MAR225 CCD detector. A complete dataset from a selenomethionine-derivatised crystal was similarly collected at the absorption peak wavelength of selenium on a Pilatus 6 M pixel detector (from Dectris) at beam line XALOC of the ALBA synchrotron (Cerdanyola, Barcelona^68^). Crystals were tetragonal and contained one protein molecule in the asymmetric unit (solvent content 57%, V_M_ = 2.9Å^3^/Da^69^). Diffraction data were processed with programs XDS^70^ and XSCALE^71^, and transformed with XDSCONV to formats suitable for the PHENIX^72^ and CCP4 suites^73^ of programs. Table 3 provides essential data-processing statistics.

### Structure solution and refinement

The structure of PorZ was solved by single-wavelength anomalous diffraction with data collected at the selenium absorption peak wavelength from the selenomethionine-derivatized crystal, applying the AUTOSOL protocol from the PHENIX package^74^. The program found 10 of the 12 theoretical selenium sites *plus* seven minor sites corresponding to surface ions, as determined a posteriori, which resulted from the crystallization conditions containing zinc and cacodylate (see the previous section). Phasing with all these sites identified P4_3_2_1_2 as the correct enantiomorphic space group and yielded an initial figure of merit of 0.26. Subsequent automatic density modification and model building produced a partial model of 373 (mostly alanine) residues in 72 chains, which showed an overall model-map correlation of 0.34 and an R_free_ value of 0.46. The resulting Fourier map enabled us to perform manual model building with the COOT program^75^, which was assisted by a homology model of PorZ obtained by threading with the LOMETS server (http://zhanglab.ccmb.med.umich.edu/LOMETS^76^). A more complete partial model was obtained and refined against the diffraction data of the selenomethionine derivative to 3.1 Å resolution with the PHENIX^77^ and BUSTER/TNT^78^ programs. The refined partial model was subsequently used to solve the native structure with data to 2.9 Å resolution by Fourier synthesis with PHENIX. Given the anisomorphism in cell axes a and b between the native and derivative data (see Table 3), these calculations included rigid-body refinement and simulated annealing, in addition to individual coordinate, translation/libration/screw-motion (TLS) and grouped thermal-displacement-parameter refinement. Thereafter, careful manual model building alternated with two cycles of automatic model building and refinement with the AUTOBUILD routine of PHENIX^79^. Finally, additional manual model building was alternated with regular crystallographic refinement using PHENIX and BUSTER/TNT, including TLS refinement, over several cycles until the final model of PorZ was obtained. This consisted of residues G^29^-R^776^ *plus* a strongly bound calcium ion. In addition, eight zinc ions, four calcium ions, one chloride ion, one cacodylate ion, one tetraethylene glycol, one diethylene glycol and four glycerol molecules arising from the crystallization and cryo-protection conditions were tentatively assigned on the surface of the molecule in addition to 43 unambiguous solvent molecules. See Table 3 for the final refinement and model quality statistics.

### Bioinformatics and statistical analyses

Structural similarity searches were performed with DALI^80^, and figures were prepared with the CHIMERA program^81^. Structure superpositions were performed with the SSM routine^82^ within COOT. The final structure of PorZ was validated with MOLPROBITY^83^ and deposited with the Protein Data Bank (PDB) at www.pdb.org (access code 5M11). Quantitative RT-PCR results were tested for normality distribution, and differences were analysed using Student’s t-test with SPSS v.16 software. Differences in enzyme activities and PorZ surface exposure in mutants in comparison to the wild-type parental strain were analyzed with GraphPad Prism 7 software (GraphPad Software, CA, USA) using one-way ANOVA with Bonferroni’s correction. *P*-values below 0.05 were considered significant.

## Additional Information

**How to cite this article**: Lasica, A. M. *et al.* Structural and functional probing of PorZ, an essential bacterial-surface component of the type-IX secretion system of human oral-microbiomic *Porphyromonas gingivalis. Sci. Rep.* **6**, 37708; doi: 10.1038/srep37708 (2016).

**Publisher's note:** Springer Nature remains neutral with regard to jurisdictional claims in published maps and institutional affiliations.

## Supplementary Material

Supplementary Information

## Figures and Tables

**Table 1:** *P. gingivalis* strains used in this study.

Strain	Relevant genotype	Source
W83	Wild type	Reference strain
HG66	Wild type	Reference strain
ΔPorZ	*porZ* (NCBI: PG_RS07070; old locus PG1604)(Em^r^)	This study
PorZ^+^	*porZ*^*+*^(Em^r^)	This study
ΔPorU	*porU* (NCBI: PG_RS00120; old locus PG0026)(Em^r^)	This study
PorU^C690A^	*porU*p.C690A(Em^r^)	This study
ΔPorN	*porN* (NCBI: PG_RS01305; old locus PG0291)(Em^r^)	This study
*PorZ modifications*		
F677i8H	*porZ*p.F677_insHHHHHHHH Q678(Em^r^)	This study
Q678i6H	*porZ*p.Q678_insHHHHHH_T679(Em^r^)	This study
D690i6H	*porZ*p.D690_ insHHHHHH_G691(Em^r^)	This study
I770i6H	*porZ*p.I770_ insHHHHHH_R771(Em^r^)	This study
R776i8H	*porZ*p.R776insHHHHHHHH(Em^r^)	This study
S683 > 6 H	*porZ*p.G684H;S685H;A686H;S687H;E688H;L669H(Em^r^)	This study
A686 > 6 H	*porZ*p.S687H;E688H;L689H;D690H;G691H;V692H(Em^r^)	This study
L689 > 6 H	*porZ*p.D690H;G691H;V692H;Y693H;V694H;Y695H(Em^r^)	This study
I770 > 6 H	*porZ*p.R771H;F772H;A773H;V774H;I775H;R776H(Em^r^)	This study

**Table 2:** *E. coli* strains and plasmids used in this study.

Strain	Relevant genotype	Source
TOP10	F- *mcrA* Δ(*mrr-hsd*RMS-*mcr*BC) Φ80*lac*ZΔM15 Δ *lac*X74 *rec*A1 *ara*D139 Δ(*araleu*)7697 *gal*U *gal*K *rps*L (StrR) *end*A1 *nup*G	Invitrogen
NEB^®^ 5-alpha	*fhuA2 Δ(argF-lacZ)U169 phoA glnV44 Φ80 Δ(lacZ)M15 gyrA96 recA1 relA1 endA1 thi-1 hsdR17*	New England Biolabs
BL21 (DE3)	*fhuA2 [lon] ompT gal (λ DE3) [dcm] ∆hsdS λ DE3* *=* *λ sBamHIo ∆EcoRI-B int::(lacI::PlacUV5::T7 gene1) i21 ∆nin5*	Invitrogen
**Plasmid**	**Relevant features**	**Source**
pUC19	*E. coli* cloning vector; Ap^r^	Thermo Scientific
pCR-BluntII-TOPO	*E. coli* cloning vector; Km^r^, Zeo^r^	Invitrogen
pGEX-6P-1	*E. coli* expression vector, PreScission Protease cleavage site between N-terminal GST tag and target protein; Ap^r^	GE Healthcare
pVA2198	*E. coli*-*Bacteroides* shuttle vector, source of *ermF*-ermAM cassette; Sp^r^	55
pT-COW	*E. coli*-*Bacteroides* shuttle vector, Tc^r^	58
p1604AeB-D	Plasmid for *porZ* deletion mutagenesis, derivative of pUC19	This study
p291AeB-C	Plasmid for *porN* deletion mutagenesis, derivative of pUC19	This study
pPorU/pUC19/Erm	Plasmid for *porU* deletion mutagenesis, derivative of pUC19	This study
pT-COW-porZ	Plasmid for *porZ* complementation, derivative of pT-COW	This study
p1604CeB-H	Master plasmid for PorZ modifications; derivative of pUC19	This study
pAT1	Plasmid for Q678i6H mutagenesis in PorZ, derivative of p1604CeB-H	This study
pAG1	Plasmid for S683 > 6 H mutagenesis in PorZ, derivative of p1604CeB-H	This study
pAL5	Plasmid for I770i6H mutagenesis in PorZ, derivative of p1604CeB-H	This study
pAL11	Plasmid for A686 > 6 H mutagenesis in PorZ, derivative of p1604CeB-H	This study
pAL12	Plasmid for L689 > 6 H mutagenesis in PorZ, derivative of p1604CeB-H	This study
pAL13	Plasmid for D690i6H mutagenesis in PorZ, derivative of p1604CeB-H	This study
p1604M1	Plasmid for I770 > 6 H mutagenesis in PorZ, derivative of p1604CeB-H	This study
p1604M2	Plasmid for F677i8H mutagenesis in PorZ, derivative of p1604CeB-H	This study
p1604M3	Plasmid for R776i8H mutagenesis in PorZ, derivative of p1604CeB-H	This study
pPorU-E	Master plasmid for PorU modification, derivative of pUC19	This study
pPorU/C690A	Plasmid for C690A mutagenesis in PorU, derivative of pPorU-E	This study
pGEX-6P-1/PorZ	Plasmid for PorZ purification from *E. coli*, derivative of pGEX-6P-1	This study

**Table 3:** Crystallographic data.

*Dataset*	*PorZ (Se absorption peak)*	*PorZ (native)*
Space group	P4_3_2_1_2	P4_3_2_1_2
Cell constants (a, b, c, in Å)	113.4, 113.4, 139.6	115.5, 115.5, 139.9
Wavelength (Å)	0.9793	0.8726
No. of measurements/unique reflections	144,224/31,485^f^	260,647/21,600
Resolution range (Å) (outermost shell)^a^	44.0–3.10 (3.18 –3.10)	48.5–2.90 (3.05–2.90)
Completeness (%)	99.7 (98.6)	99.9 (99.7)
R_merge_^b^	0.146 (0.976)	0.108 (0.913)
R_r.i.m._ [=R_meas_] c/CC(^1^/_2_) c	0.165 (1.206)/0.993 (0.584)	0.113 (0.953)/0.999 (0.837)
Average intensity d	10.8 (1.8)	20.0 (3.4)
B-Factor (Wilson) (Å^2^)/Aver. multiplicity	63.0/4.6 (2.8)	63.3/12.1 (12.1)
Number of Se-atom sites used for phasing	10 (out of 12)	
Resolution range used for refinement (Å)		48.5–2.90
No. of reflections used (test set)		20,876 (723)
Crystallographic R_factor_ (free R_factor_)^b^		0.183 (0.238)
No. of protein atoms/solvent molecules/ neutral ligands/ ionic ligands		5,678/43/ 1 tetraethylene glycol, 1 diethylene glycol, 4 glycerol 8 zinc, 4 calcium, 1 chloride, 1 cacodylate
*Rmsd* from target values^e^		
bonds (Å)/angles (°)		0.010/1.22
Average B-factors (Å^2^)		74.8
All-atom contacts and geometry analysis^e^		
Residues		
in favored regions/outliers/all residues		701 (94.0%)/1/746
with poor rotamers/bad bonds/bad angles		30 (5.0%)/0/0
with Cβ deviations >0.25 Å/clashscore		0/9.13 (97^th^ percentile)
MolProbity score		2.42 (94^th^ percentile)

^a^Values in parenthesis refer to the outermost resolution shell.

^b^For definitions, see Table 1 in ref. 85.

^c^For definitions, see refs 86 and 87.

^d^Average intensity is <I/σ(I)> of unique reflections after merging according to the XDS program^70^.

^e^According to MOLPROBITY^83^,^88^. ^f^ Friedel mates were kept separately.
