# Supplementary material for: Structural and functional probing of PorZ, an essential bacterial surface component of the type-IX secretion system of human oral-microbiomic Porphyromonas gingivalis
Source: Sci Rep. 2016 Nov 24;6:37708. doi: 10.1038/srep37708 (PMC5121618; doi:10.1038/srep37708)
Supplement: Supplementary Information [file srep37708-s1.doc]

**Structural and functional probing of PorZ, an essential bacterial surface component of the type-IX secretion system of human oral-microbiomic *Porphyromonas gingivalis*.**

Anna M. Lasica, Theodoros Goulas, Danuta Mizgalska, Xiaoyan Zhou, Iñaki de Diego, Mirosław Ksiazek, Mariusz Madej, Yonghua Guo, Tibisay Guevara, Magdalena Nowak, Barbara Potempa, Apoorv Goel, Maryta Sztukowska, Apurva T. Prabhakar, Monika Bzowska, Magdalena Widziolek, Ida B. Thøgersen, Jan J. Enghild, Mary Simonian, Arkadiusz W. Kulczyk, Ky-Anh Nguyen, Jan Potempa and F. Xavier Gomis-Rüth

**SUPPLEMENTARY TABLES AND FIGURES**

| **Supplementary Table S1.** **Identification of CTD-proteins in the growth medium of wild-type *P. gingivalis* and mutant ΔPorZ.** | | | | | | | | | | |
| --- | --- | --- | --- | --- | --- | --- | --- | --- | --- | --- |
|  | | **Wild-type *P. gingivalis* W83** | | | | | **ΔPorZ** | | | |
| Gel slide^(a)^ | Locus | | Description | Mol. mass  [kDa] | | Mascot score | Locus | Description | Mol. mass  [kDa] | Mascot score |
| **1-3** | Molecular mass range: >150 kDa | | | | | | | | | |
|  | - | | - | - | | - | PG1844 | Kgp^(b)^ | 187.8 | 607^(c)^ |
|  |  | |  |  | |  | PG1837 | HagA | 233.3 | 529 |
|  |  | |  |  | |  | PG2024 | RgpA | 185.6 | 453 |
| **4** & **5** | Molecular mass range: 80 – 150 kDa | | | | | | | | | |
|  | PG0410 | | C25 peptidase | 144.0 | | 255 | **PG0026** | **PorU sortase**^(d)^ | **124.5** | **1,340** |
|  | PG0026 | | PorU sortase | 124.5 | | 49 | PG1844 | Kgp | 187.8 | 1,191 |
|  |  | |  |  | |  | PG2024 | RgpA | 185.6 | 1,007 |
|  |  | |  |  | |  | PG1837 | HagA | 233.3 | 760 |
|  |  | |  |  | |  | **PG0411** | **putative HA** | **103.6** | **141** |
| **6** & **7** | Molecular mass range: 60 – 90 kDa | | | | | | | | | |
|  | PG0232 | | CPG70 | 91.5 | | 57 | **PG0232** | **CPG70** | **91.5** | **2,806** |
|  |  | |  |  | |  | PG0506 | RgpB | 80.8 | 2,097 |
|  |  | |  |  | |  | PG1844 | Kgp | 187.8 | 1,750 |
|  |  | |  |  | |  | PG2024 | RgpA | 185.6 | 1,720 |
|  |  | |  |  | |  | PG1844 | Kgp | 187.8 | 1,050 |
|  |  | |  |  | |  | PG1837 | HagA | 233.3 | 889 |
|  |  | |  |  | |  | PG0553 | LysP | 102.5 | 59 |
| **8** & **9** | Molecular mass range: 40 – 70 kDa | | | | | | | | | |
|  | PG2024 | | RgpA | 185.6 | | 735 | **PG1424** | **PPAD** | **61.7** | **2,150** |
|  | PG1424 | | PPAD | 61.7 | | 686 | **PG0495** | **Uncharacterised** | **53.9** | **2,105** |
|  | PG1844 | | Kgp | 187.8 | | 587 | PG0506 | RgpB | 80.8 | 2,068 |
|  | PG0506 | | RgpB | 80.8 | | 430 | PG2024 | RgpA | 185.6 | 1,636 |
|  | PG0350 | | Internalin-like | 52.6 | | 152 | **PG0350** | **Internalin-like** | **52.6** | **933** |
|  | PG1427 | | PrtT | 93.1 | | 68 | PG1837 | HagA | 233.3 | 930 |
|  | PG0654 | | Uncharacterised | 44.8 | | 63 | **PG1798** | **Antigen PG99** | **45.7** | **155** |
|  |  | |  |  | |  | PG1427 | PrtT | 93.1 | 134 |
|  |  | |  |  | |  | PG0026 | PorU sortase | 124.5 | 86 |
|  |  | |  |  | |  | PG2102 | TapA | 61.1 | 56 |
|  |  | |  |  | |  | PG0350 | Internalin-like | 52.6 | 56 |
| **10** & **11** | Molecular mass range: 30 – 50 kDa | | | | | | | | | |
|  | PG2024 | | RgpA | | 185.6 | 506 | PG2024 | RgpA | 185.6 | 4,024 |
|  | PG1844 | | Kgp | | 187.8 | 248 | PG1844 | Kgp | 187.8 | 2,567 |
|  | PG1837 | | HagA | | 233.3 | 240 | **PG1374** | **Internalin** | **47.15** | **2,282** |
|  | PG0410 | | C25 peptidase | | 144.0 | 255 | **PG0654** | Uncharacterised | 44.8 | 1,315 |
|  | PG0654 | | Uncharacterised | | 44.8 | 214 | PG0506 | RgpB | 80.9 | 862 |
|  | PG1374 | | Internalin | | 47.1 | 152 | **PG1030** | **Uncharacterised** | **50.1** | **688** |
|  |  | |  | |  |  | **PG0616** | **HBP35** | **37.5** | **642** |
|  |  | |  | |  |  | PG1837 | HagA | 233.3 | 537 |
|  |  | |  | |  |  | **PG2216** | **Uncharacterised** | **61.3** | **214** |
| ^(a)^ The pattern of gel slicing is shown in supplementary Figure S1.  ^(b)^ Fragments of HagA, RgpA, RgpB, PPAD, CPG70, and PorU were detected in different molecular mass zones, but no CTD-  derived peptides were detected. Only fragments with the Mascot score >500 are listed.  ^(c)^ The molecular-mass zones overlap, so the score is the sum of scores from adjacent gel slides. Therpotein is assigned to the   zone with higher score.  ^(d)^ CTD-proteins, for which CTD-derived peptides were detected, are shown in bold. | | | | | | | | | | |

| **Supplementary Table S2.** **Primers used in this study**. | |
| --- | --- |
| **Primer name** | **Sequence (5’->3’)** |
| **p1604AeB-D plasmid for deletion of *porZ*** | |
| PG1604FrANdeIF | AAGGTCATATGCGTGATACCCTCGTAGTG |
| PG1604FrASmaR | AAATTCCCGGGCCAAAGCACGATGACTG |
| ermFAMSmaIF | ATTACCCGGGATAGCTTCCGCTATTGC |
| ermFAMSalIR | GCAGGTCGACTCTAGAGGATCCC |
| PG1604FrBXbaIF | GATCTAGAAGTGATTCGCTGACACAC |
| PG1604FrBSalIR | AATTGTCGACTCACGTATGTCCAGTTCTTG |
| **p1604CeB-H master plasmid for PorZ mutagenesis** | |
| PG1604FrANdeIF2 | CAACACATATGGCAAAATAAGATTCAGCCTCTT |
| PG1604FrCSmaR | TATATCCCGGGTGTGTCAGCGAATCACT |
| ermFAMSmaIF | ATTACCCGGGATAGCTTCCGCTATTGC |
| ermFAMSalIR | GCAGGTCGACTCTAGAGGATCCC |
| PG1604FrBXbaIF | GATCTAGAAGTGATTCGCTGACACAC |
| PG1604FrBSalIR | AATTGTCGACTCACGTATGTCCAGTTCTTG |
| **PorZ R776i8H mutagenesis primers yielding p1604M3 plasmid** | |
| 1604M1Fs | TGACACAcccgggATAGCTTCC |
| 1604M3Rs | GCGAATCACTGCGAAGCGA |
| 1604M3Ft | CACCATcaccatcaccatcaccatTGACACAcccgggATAGCTTCC |
| 1604M3Rt | ATGGTGatggtgatggtgatggtgGCGAATCACTGCGAAGCGA |
| **PorZ F677i8H mutagenesis primers yielding p1604M2 plasmid** | |
| 1604M2Fs | caaacgggtacggggagtgg |
| 1604M2Rs | gaacgtcatcagtccgtccgc |
| 1604M2Ft | CACCATcaccatcaccatcaccatcaaacgggtacggggagtgg |
| 1604M2Rt | ATGGTGatggtgatggtgatggtggaacgtcatcagtccgtccgc |
| **PorZ I770>6H mutagenesis primers yielding p1604M1 plasmid** | |
| 1604M1Fs | TGACACAcccgggATAGCTTCC |
| 1604M1Rs | AATTAGTTTGGACTTTTTCGATACCG |
| 1604M1Ft | caccatcaccatcaccatTGACACAcccgggATAGCTTCC |
| 1401M1Rt | ATGGTGatggtgatggtgAATTAGTTTGGACTTTTTCGATACC |
| **PorZ Q678i6H mutagenesis primers yielding pAT1 plasmid** | |
| PG1604_i_Q678_FwA | CATCACCATCACCATCACACGGGTACGGGGAGTGGATCAGC |
| PG1604_i_Q678_RevB | GTGATGGTGATGGTGATGTTGGAACGTCATCAGTCCGTCCG |
| PG1604_nt_Q678_FwC | ACGGGTACGGGGAGTGGATCAGC |
| PG1604_nt_Q678_RevD | TTGGAACGTCATCAGTCCGTCCG |
| **PorZ D690i6H mutagenesis primers yielding pAL13 plasmid** | |
| PG1604_i_D690_FwA | CATCACCATCACCATCACGGCGTCTATGTATACCCCAATCC |
| PG1604_i_D690_RevB | GTGATGGTGATGGTGATGGTCCAGTTCGGAAGCTGATCC |
| PG1604_nt_D690_FwC | GGCGTCTATGTATACCCCAATCCG |
| PG1604_nt_D690_RevD | GTCCAGTTCGGAAGCTGATCCACTC |
| **PorZ I770i6H mutagenesis primers yielding pAL5 plasmid** | |
| PG1604_s_I770_FwA | CATCACCATCACCATCACCGCTTCGCAGTGATTCGCTGAC |
| PG1604_s_I770_RevB | GTGATGGTGATGGTGATGAATTAGTTTGGACTTTTTCGATAC |
| PG1604_nt_I770_FwC | CGCTTCGCAGTGATTCGCTGAC |
| PG1604_nt_I770_RevD | AATTAGTTTGGACTTTTTCGATAC |
| **PorZ S683>6H mutagenesis primers yielding pAG1 plasmid** | |
| PG1604_s_S683_FwA | CATCACCATCACCATCACGACGGCGTCTATGTATACC |
| PG1604_i_s_S683_RevB | GTGATGGTGATGGTGATGACTCCCCGTACCCGTTTGG |
| PG1604_nt_S683_FwC | GACGGCGTCTATGTATACCCCAATCC |
| PG1604_S683_RevD | ACTCCCCGTACCCGTTTGGAC |
| **PorZ A686>6H mutagenesis primers yielding pAL11 plasmid** | |
| PG1604_s_A686_FwA | CATCACCATCACCATCACTATGTATACCCCAATCCGCTAAG |
| PG1604_s_A686_RevB | GTGATGGTGATGGTGATGAGCTGATCCACTCCCCGTACC |
| PG1604_nt_A686_FwC | TATGTATACCCCAATCCGCTAAGG |
| PG1604_nt_A686_RevD | AGCTGATCCACTCCCCGTACC |
| **PorZ L689>6H mutagenesis primers yielding pAL12 plasmid** | |
| PG1604_s_L689_FwA | CATCACCATCACCATCACCCCAATCCGCTAAGGCCGGAATATC |
| PG1604_s_L689_RevB | GTGATGGTGATGGTGATGCAGTTCGGAAGCTGATCC |
| PG1604_nt_L689_FwC | CCCAATCCGCTAAGGCCGGAATATC |
| PG1604_nt_L689_RevD | CAGTTCGGAAGCTGATCCACTC |
| **p291AeB-C plasmid for deletion of *porN*** | |
| PG291FrBXbaIF | GCGTCTAGAGTATTCGGACTGCATCT |
| PG291FrBPstIR | ATTACTGCAGCGTCTGAATCGTGCAAA |
| ermFAMSmaIF | ATTACCCGGGATAGCTTCCGCTATTGC |
| ermFAMXbaIR | GCTCTAGACGAAGCTGTCAGTAGTATACC |
| PG291FrANdeIF | TACCACATATGACTGTAGCAGCCATTACAC |
| PG291FrASmaIR | TAATTCCCGGGCTCCTATGACTGCTTTGA |
| **pPorU/pUC19/Erm plasmid for deletion of *porU*** | |
| PorUFrag5EcoRI | ACTTGAATTCAGTCGATGGCCGATGAGATGATAG |
| PorUFrag5KpnI | AATAGGTACCATCTGAGAAATAGCTTATTTG |
| PorUFrag3BamHI | AATAGGATCCAATAGCCGAAGCAGTTCTGCCG |
| PorUFrag3PstI | TTCCTGCAGATTCTTCCTTGAGTCCTCC |
| ErmFAMKpnI | GTACCCCCGATAGCTTCCGCTATTGCTTTTTTG |
| ermFAMBamHI | GGATCCCCGAAGCTGTCAGTAGTATACCTAATAATTTA |
| **pPorU-E master plasmid for PorU mutagenesis** | |
| Pg26_AR | CAGTCTAGAGGCTATTGTCCTACCACGATCAT |
| Pg26_AF | CATGAATTCTTCCCTGACAGCATCG |
| Pg26_BR | TCGAAGCTTGCTAAGTTCAAGGTTGAA |
| Pg26_BF | TGACTGCAGCAATAGCCGAAGCAGTTCTGC |
| Pg26_ER | TCACTGCAGTTACGTTTCCGCTCCATC |
| Pg26_EF | TGCTCTAGAATAGCTTCCGCTATTGCTTT |
| **PorU^C690A^ mutagenesis primers yelding pPorU/C690A plasmid** | |
| 26C667AFt | TTACTGCCACGGCCGACTTTGCCAACTATGACAGTCAGA |
| P26C667AFs | TTGCCAACTATGACAGTCAGA |
| 26C667ARt | AGTCGGCCGTGGCAGTAATCCAAATGGGCATATGCTTAT |
| P26C667ARs | TCCAAATGGGCATATGCTTAT |
| **Primers for recombinant PorZ production from pGEX-6P-1/PorZ plasmid** | |
| F1604Sma | AATTCCCGGGTCAAGAGGAAGGTATTTGGAATAC |
| R1604Not | AATTGCGGCCGCTTAGCGAATCACTGCGAA |
| **pT-COW-porZ plasmid for complementation of PorZ** | |
| pT-COWporZForSalI | CTCGTCGACATCCACATAAGCTCAACGAGATTC |
| pT-COWporZRevNheI | ACTGCTAGCTTGGACACGATGGCTAAACTGA |

| **qPCR primers and probes**. | |
| --- | --- |
| ***porT*** | |
| PgPorTTMF | AGAGTCGCTCTCTTTTGCCTGAT |
| PgPorTTMR | TGGAATCCAAGATGATAGCGTTTGTAGTC |
| fPgPorTProbeBFQ | CCTGGGCCGGACGTACGCTGTCG |
| ***sov*** | |
| SovTMF | CAGCGCAAAACGATGTTCGG |
| SovTMR | TGGTCGTCAGCGGCATTTC |
| FamSovBH1 | CACCCTCGGCGGCACCTTCATGCAC |
| ***porU*** | |
| PG26TMF | ACCCAATCAAAACCTGCATGGA |
| PG26TMR | GCAACACGACCAAAACCTTCAG |
| fPG26bh1 | ACTCAGGCGCTCCTCCTTGAGGCTGA |
| ***lptO*** | |
| PG27TMF | CGGGTATGAGGCTGCACTC |
| PG27TMR | AGTCCCCATGTAATCTCACGGAA |
| FamPG27BH1 | CCTTGAGTCCTCCCGGCGCATCACCG |
| ***porN*** | |
| PG291TMF | GGTCAGTATCTCGTCCGTTGC |
| PG291TMR | AGTCGTTTGTTCCACCTCGGTA |
| FamPG291BH1 | CCGCTCTCCGCAAGTGGGTCGAGC |
| ***porO*** | |
| PG602TMF | GGCAGGTGCTCAACAAGAGA |
| PG602TMR | GGGATTGTCGTCTACGATGGTGAT |
| FamPG602BH1 | TGCCTCCGGCAGCCAAAGCCTGT |
| ***porW*** | |
| PG809TMF | GGAGCATTCGATCAGTGGCT |
| PG809TMR | TCGGAGCGAAAGCATCCTTG |
| fPG809bh1 | TGACCTCTCTCCCGACCCCACGCA |
| ***rgpB*** | |
| RgpBTMF | CGATCGTAGCATTCTCCTCTCTGTTG |
| RgpBTMR | CAGCGGAGAGCAGTCGTACT |
| fRgpBProbeIBFQ | CCGGCAGAGCGCGGTCGCAAC |
| ***kgp*** | |
| KgpTMF | AGTAGGAACGACAAACGCCTCTA |
| KgpTMR | AGTGTCACCAACCAAAGCCA |
| fKgpBH1 | AGACCGGAGCAGCACTAGCTGCCAATCCA |
| ***cpg70*** | |
| CpgTMF | AGCGTCCAATTCCAAGATCAAACG |
| CpgTMR | TACGAGCGGATTCTGCTCTGT |
| fCpgPrBH1 | ACGTTCGAAGGCGGACAGCCTGCCAT |
| ***r16s*** | |
| Pg16sTMF | TCGGTAAGTCAGCGGTGAAAC |
| Pg16sTMR | GCAAGCTGCCTTCGCAATC |
| Cy5Pg16sPrIBRQ | CCGCCACTGAACTCAAGCCCGGCA |
| ***gyrA*** | |
| GyrATMF2 | GGGATCGGATACTCGCGAAGA |
| GyrATMR | GGATTTCATACACCTTCAGCCAATAGCA |
| hGyrAProbeBFQ | ACTCGGCCTCCATGCACGCCACC |

**
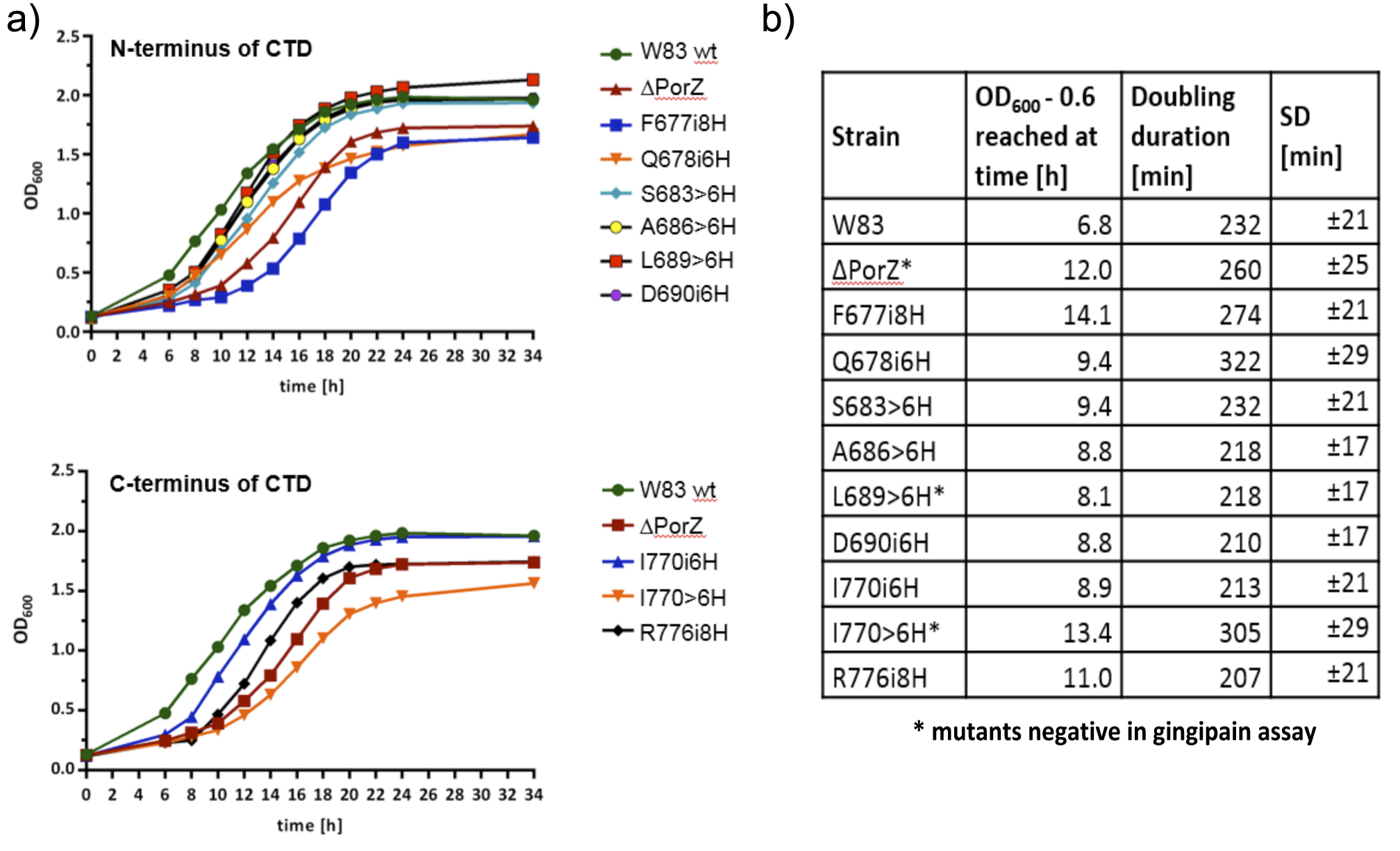
**

**Supplementary Figure S1. Growth curves and doubling times of *P. gingivalis* strains.** (**a**) Growth curves of strains with insertions/substitutions of oligohistidines in PorZ at the linker sequence preceding CTD (*top panel*) and at the C-terminus (*bottom panel*). (**b**) Doubling times of PorZ mutants. No significant differences in the final growth rates were observed. For some mutant strains (ΔPorZ, F677i8H, and I770>6H), the exponential phase was reached after a delay, which correlated with extended doubling duration. Initial starter cultures were grown under appropriate antibiotic selection, but subsequent passage culture for growth kinetics was carried out without antibiotic supplementation. Starting cultures were grown freshly (24 h) until OD_600_=1.6-1.8 and then diluted to OD_600_=0.1 with preheated eTSB medium. Then, 5 ml of each culture were transferred into individual glass screw-cap test tubes (13x100 mm), which were incubated at 37°C in an anaerobic chamber for 6 h, capped tightly, and moved into a water bath (37°C) outside the anaerobic chamber. At predetermined time points, tubes were vortexed and OD_600_–values were recorded using a SpectraMax Plus spectrophotometer. Growth curves for each clone were determined for three replicate samples. Doubling times were calculated from the growth curves at three points (OD_600_= 0.5, 0.55 and 0.6) using a linear regression function of one phase exponential decay calculated with the GraphPad Prism 7 program (GraphPad Software, CA, USA).


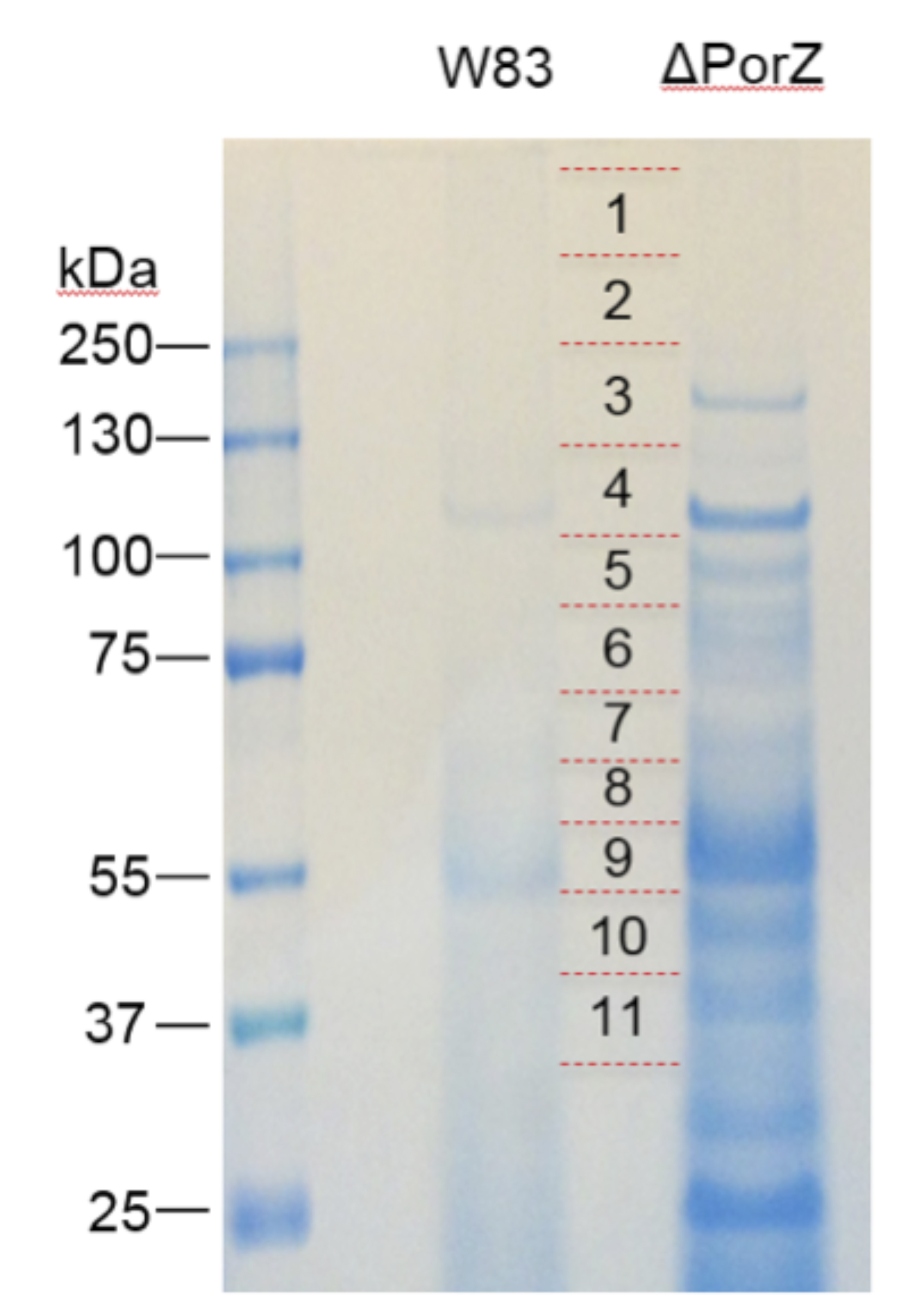


**Supplementary Figure S2. Secreted proteins of mutant ΔPorZ.** SDS-PAGE profile of proteins in 20-fold concentrated, particle-free, spent growth media of wild-type *P. gingivalis* (W83) and ΔPorZ mutant at approximately the same stage of growth. Indicated sections of the gel were subjected to finger-printing analysis to identify proteins released into the medium (see **Supplementary Table S1**).


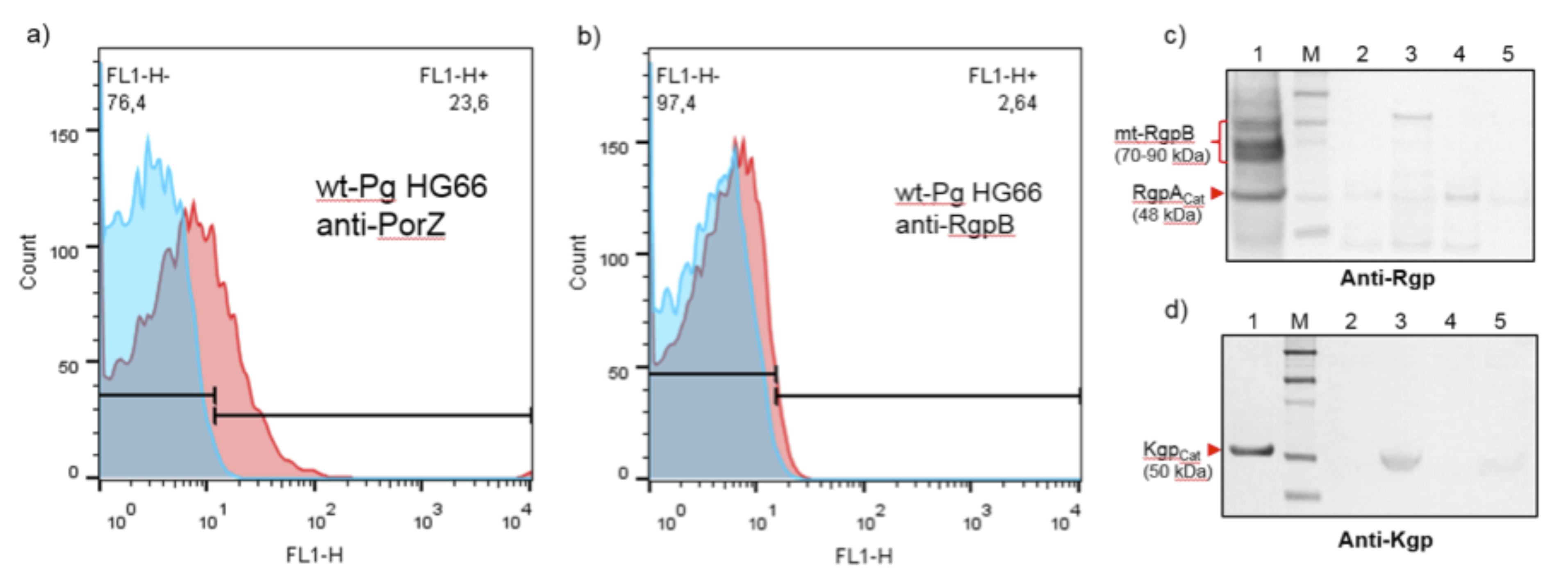


**Supplementary Fig. S3. Surface location of PorZ in the *P. gingivalis* HG66 strain.** The protein is located on the cell-surface of a *P. gingivalis* strain HG66 variant deficient in anionic-lipopolysaccharide (A-LPS) synthesis (**a**) ^1^, which lacks cell-surface-associated gingipains (**b**) ^2^. In contrast to strain HG66, strain W83 retains gingipains tightly anchored in the outer membrane *via* A-LPS covalently attached to gingipain molecules, so they withstand cell washing with water or diluted detergents**.** HG66 strain cells were incubated with mouse anti-PorZ polyclonal antibodies (a) and rabbit anti-RgpB monoclonal antibodies (b), stained with fluorescein-labeled rabbit anti-mouse antibodies and subjected to FACS analysis. Representative histograms of experiments repeated three times (on different cultures) in triplicates are shown. (**c** and **d**) Bacterial cells of *P. gingivalis* strain W83 were washed with PBS and then suspended in distilled water (*lane 2*), 0.0007 % Tween (*lane 3*), 0.04 % sarcosyl (*lane 4*), and 0.02 % SDS (*lane 5*). After 10 min of gentle steering, cells were removed by centrifugation. The presence of Rgp (**c**) and Kgp (**d**) in cell pellets (*lane 1*) and in 30-fold concentrated supernatants (*lanes 2-5*) was visualized by Western-blot analysis. Concentrations (%) represent 1/10 of the respective critical micelle concentration of each detergent. M - MagicMark™ XP Western Protein Standard.

1 Shoji, M., Sato, K., Yukitake, H., Naito, M. & Nakayama, K. Involvement of the Wbp pathway in the biosynthesis of *Porphyromonas gingivalis* lipopolysaccharide with anionic polysaccharide. *Sci. Rep*. **4,** 5056 (2014)

2 Potempa, J., Pike, R. & Travis, J. The multiple forms of trypsin-like activity present in various strains of *Porphyromonas gingivalis* are due to the presence of either Arg-gingipain or Lys-gingipain. *Infect. Immun*. **63,** 1176-1182 (1995)

**
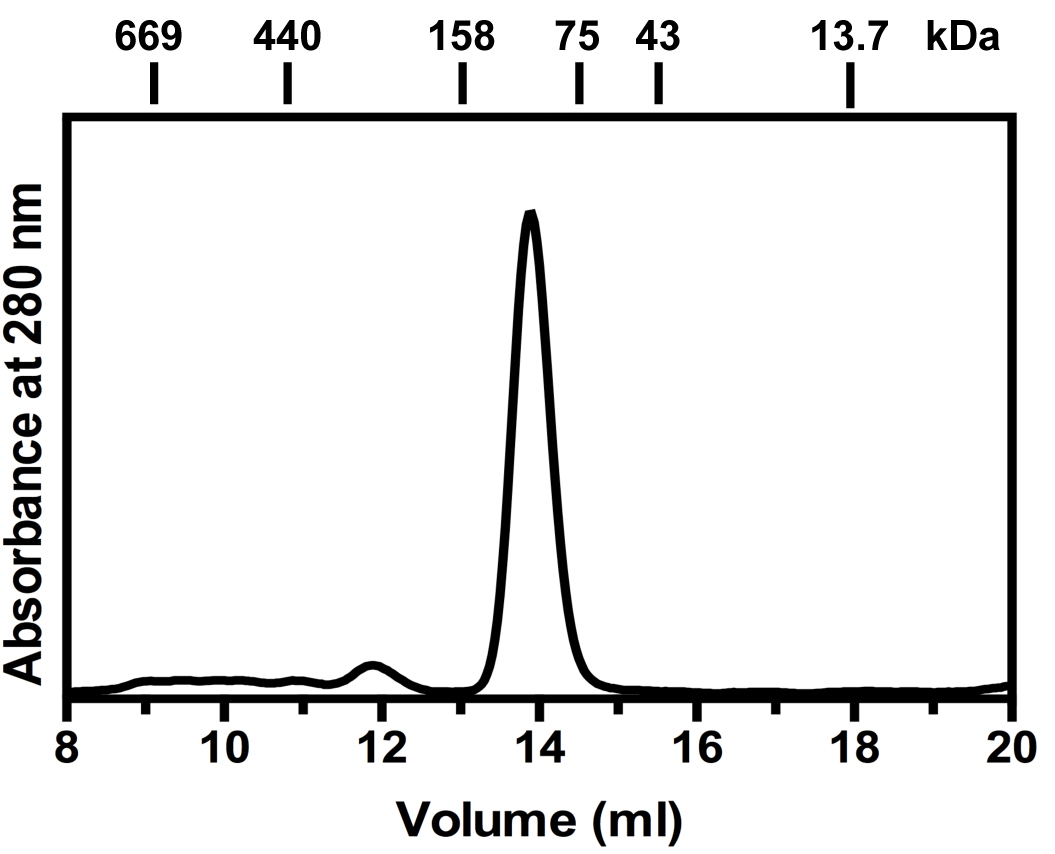
**

**Supplementary Figure S4. PorZ is a monomer.** Purified PorZ was separated on calibrated HiLoad Superdex 200 10/300 GL column (GE Healthcare Life Sciences) equilibrated in 20 mM Tris, 150 mM NaCl, pH 7.5. rPorZ eluted at 13.9 ml equivalent to the 80 kDa globular protein.


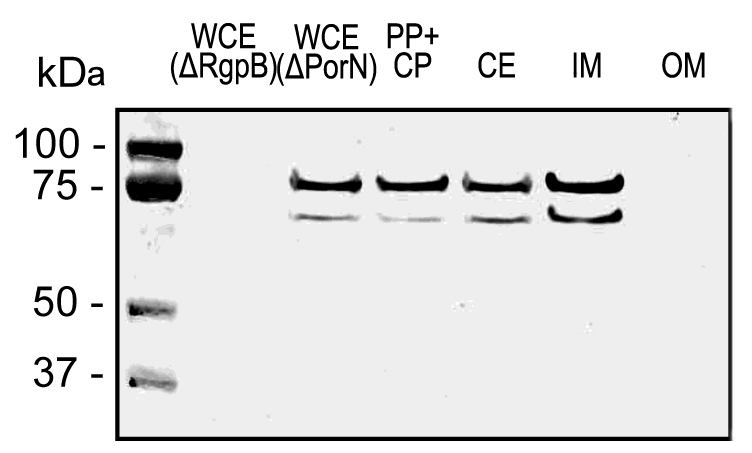


**Supplementary Figure S5. Subcellular location of RgpB in the *P. gingivalis* ΔPorN strain.** Whole-cell extract (WCE) was proportionately fractionated into periplasm and cytoplasm (PP+CP), cell envelope (CE), inner membrane (IM) and outer membrane (OM), and probed for RgpB with specific monoclonal antibodies. WCE derived from ΔRgpB was used as a negative control.
